# TDP-43 Triggers Mitochondrial DNA Release via mPTP to Activate cGAS/STING in ALS

**DOI:** 10.1016/j.cell.2020.09.020

**Published:** 2020-10-29

**Authors:** Chien-Hsiung Yu, Sophia Davidson, Cassandra R. Harapas, James B. Hilton, Michael J. Mlodzianoski, Pawat Laohamonthonkul, Cynthia Louis, Ronnie Ren Jie Low, Jonas Moecking, Dominic De Nardo, Katherine R. Balka, Dale J. Calleja, Fiona Moghaddas, Erya Ni, Catriona A. McLean, Andre L. Samson, Shiraz Tyebji, Christopher J. Tonkin, Christopher R. Bye, Bradley J. Turner, Genevieve Pepin, Michael P. Gantier, Kelly L. Rogers, Kate McArthur, Peter J. Crouch, Seth L. Masters

**Affiliations:** 1 Inflammation Division, The Walter and Eliza Hall Institute of Medical Research, Parkville, VIC 3052, Australia; 2 Centre for Dynamic Imaging, The Walter and Eliza Hall Institute of Medical Research, Parkville, VIC 3052, Australia; 3 Personalised Oncology Division, The Walter and Eliza Hall Institute of Medical Research, Parkville, VIC 3052, Australia; 4 Infection and Immunity Division, The Walter and Eliza Hall Institute of Medical Research, Parkville, VIC 3052, Australia; 5 Department of Medical Biology, University of Melbourne, Parkville, VIC 3010, Australia; 6 Department of Pharmacology and Therapeutics, University of Melbourne, Parkville, VIC 3010, Australia; 7 Florey Institute of Neuroscience and Mental Health, University of Melbourne, Parkville, VIC 3010, Australia; 8 Department of Immunology and Allergy, The Royal Melbourne Hospital, Parkville, VIC 3052, Australia; 9 Anatomical Pathology, The Alfred Hospital, Melbourne, VIC 3004, Australia; 10 Centre for Innate Immunity and Infectious Diseases, Hudson Institute of Medical Research, Clayton, VIC, Australia; 11 Department of Molecular and Translational Science, Monash University, Clayton, VIC 3168, Australia; 12 Department of Anatomy and Developmental Biology, Monash Biomedicine Discovery Institute, Monash University, Clayton, VIC 3168, Australia; 13 Institute of Structural Biology, University of Bonn, 53127 Bonn, Germany; 14 Immunology Laboratory, Guangzhou Institute of Paediatrics, Guangzhou Women and Children’s Medical Centre, Guangzhou, Guangdong 510623, China

**Keywords:** ALS, TDP-43, cGAS, STING, cGAMP, IFN, NF-κB, mitochondria, mPTP, neurodegeneration

## Abstract

Cytoplasmic accumulation of TDP-43 is a disease hallmark for many cases of amyotrophic lateral sclerosis (ALS), associated with a neuroinflammatory cytokine profile related to upregulation of nuclear factor κB (NF-κB) and type I interferon (IFN) pathways. Here we show that this inflammation is driven by the cytoplasmic DNA sensor cyclic guanosine monophosphate (GMP)-AMP synthase (cGAS) when TDP-43 invades mitochondria and releases DNA via the permeability transition pore. Pharmacologic inhibition or genetic deletion of cGAS and its downstream signaling partner STING prevents upregulation of NF-κB and type I IFN induced by TDP-43 in induced pluripotent stem cell (iPSC)-derived motor neurons and in TDP-43 mutant mice. Finally, we document elevated levels of the specific cGAS signaling metabolite cGAMP in spinal cord samples from patients, which may be a biomarker of mtDNA release and cGAS/STING activation in ALS. Our results identify mtDNA release and cGAS/STING activation as critical determinants of TDP-43-associated pathology and demonstrate the potential for targeting this pathway in ALS.

## Highlights

• TDP-43 enters mitochondria, triggers mtDNA release via the mPTP
• TDP-43-induced cytosolic mtDNA accumulation activates the cGAS/STING pathway
• Evidence of cytoplasmic mtDNA was found in ALS patient cells and disease models
• Blocking STING prevents inflammation and neurodegeneration *in vitro* and *in vivo*

## Graphical Abstract

**Figure:**
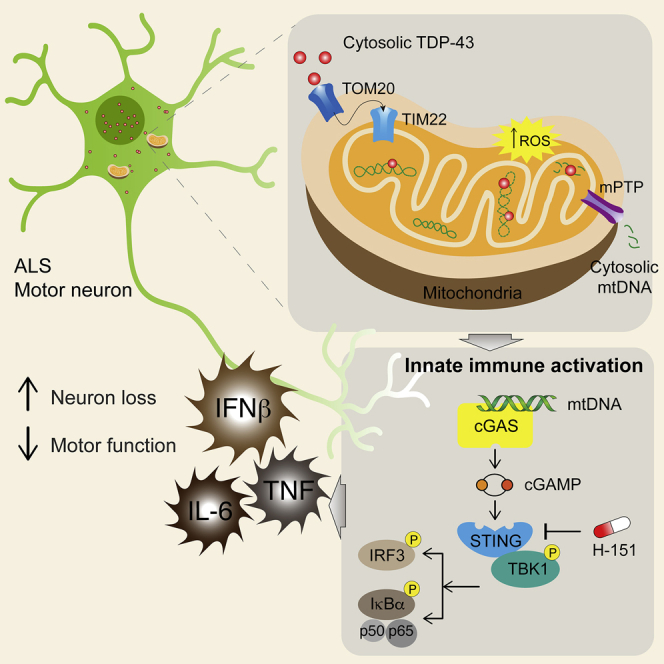

## Teaser

TDP-43 causes inflammation in ALS by stimulating mitochondrial DNA release, which is subsequently sensed by the cytosolic cGAS/STING pathway, suggesting that inhibition of cGAS/STING could help alleviate inflammation-related damage in ALS.

## Introduction

TDP-43 is a nuclear DNA/RNA binding protein that is mutated in 4% of familial amyotrophic lateral sclerosis (ALS) (Kabashi et al., 2008; Sreedharan et al., 2008); however, its cytoplasmic accumulation is also observed in neurons of almost all patients with sporadic ALS and defines the major pathological subtype of frontotemporal lobar degeneration (FTLD) (Neumann et al., 2006). Aside from two RNA binding domains, TDP-43 also encodes a nuclear localization sequence and nuclear export sequence, which mediate shuttling between the nucleus and cytosol, and a low-complexity glycine-rich region, which is where missense mutations (e.g., A315T, Q331K, or M337V) have been found to cause ALS (Kabashi et al., 2008; Sreedharan et al., 2008). The C-terminal glycine-rich domain encodes a prion-like structure that can potentiate cytoplasmic aggregation (Johnson et al., 2009). Recent studies demonstrate that ALS-associated mutations enhance TDP-43 accumulation not only in the cytoplasm but specifically within mitochondria (Magrané et al., 2014; Wang et al., 2013, 2017) and that preventing its translocation through the inner mitochondrial membrane can prevent neurotoxicity (Wang et al., 2016). This appears to be a specific way in which mislocalized TDP-43 affects homeostasis of the cell and could have more far-reaching consequences for triggering immune response pathways.

ALS, and TDP-43-mediated neurodegeneration in general, has been associated with not only hyperinflammatory responses, such as nuclear factor κB (NF-κB)-related cytokines (Egawa et al., 2012; Swarup et al., 2011; Zhao et al., 2015) but also with an elevated type I interferon (IFN) signature (Wang et al., 2011). Interestingly, these inflammatory signals precede overt symptoms in mouse models of the disease, which suggests that they participate in disease pathogenesis rather than simply acting as a marker of disease. Indeed, blockade of NF-κB appears to have some ability to reduce denervation in the neuromuscular junction and improve motor symptoms in TDP-43 (A315T) transgenic mice (Swarup et al., 2011). Despite these indications, there is currently no immune sensor proposed to detect cytoplasmic TDP-43 and trigger the inflammation observed in TDP-43 proteinopathies, laying the groundwork for this study.

## Results

### Inflammatory Signaling from TDP-43 Is Dependent on cGAS/STING

**Figure S1:**
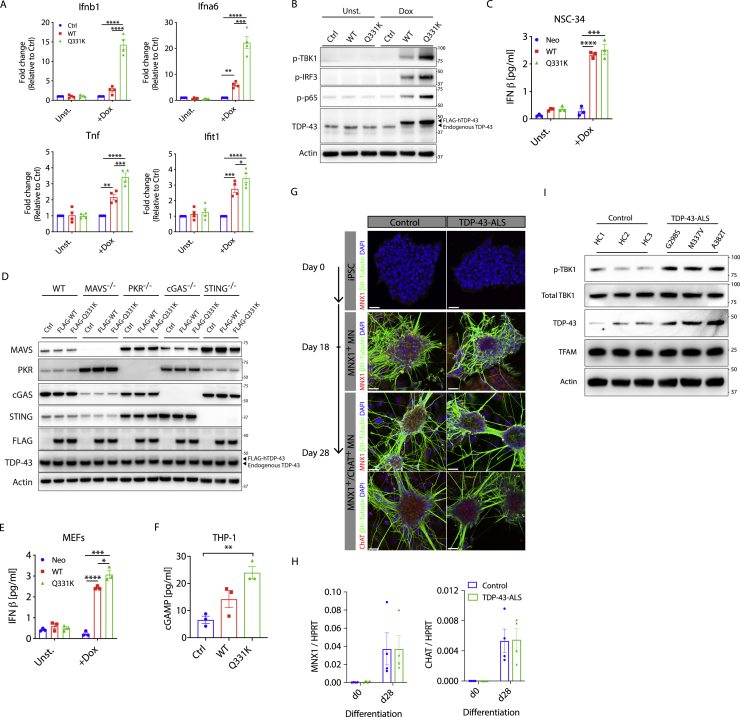
Elevated NF-κB and Type I IFN Signaling Because of TDP-43 *In Vitro*, Related to Figure 1 (A) Doxycycline (Dox inducible wild-type (WT) or ALS mutant (Q331K) TDP-43 was stably transduced into the mouse neuronal cell line NSC-34. 72 hr after TDP-43 induction, RNA was collected for qPCR of *Ifnb1*, *Ifna6*, *Ifit1* and *Tnf* or (B) cells were lysed for western blot of p-TBK1, p-IRF3, p-p65, TDP-43 and actin as control. Blots are representative of three independent experiments. (C) IFNβ ELISA was performed on the supernatant from cells in (A). (D) Representative western blot of MAVS, PKR, cGAS, STING, FLAG, TDP-43 and Actin from cells in Figure. 1A. (E) IFNβ ELISA was performed on the supernatant from MEFs after 72hrs induction of WT and Q331K TDP-43. (F) cGAMP ELISA was performed on the lysates of human THP-1 cells overexpressing TDP-43 (WT or Q331K) after 72hrs induction. (G) Images of healthy control and TDP-43-ALS patient iPSC during differentiation into premature MNX1^+^ motor neurons (day 18) and further into mature MNX1^+^/ChAT^+^ motor neurons (day 28). (red - MNX1 or ChAT, green – β3-tubulin and blue - DAPI). (scale: 40 μm). (H) *MNX1* and *CHAT* expression, measured by qPCR in undifferentiated (day 0) and differentiated iPSC-derived MNs (day 28). (I) Representative western blot of p-TBK1, total TBK1, TDP-43, TFAM and Actin for cells in (G) (lysed in 1% NP-40). Data are mean ± SEM from 3-4 independent experiments. *P value*s were calculated using one-way or two-way ANOVA. ^∗^p < 0.05, ^∗∗^p < 0.01, ^∗∗∗^p < 0.001, ^∗∗∗∗^p < 0.0001.

**Figure 1:**
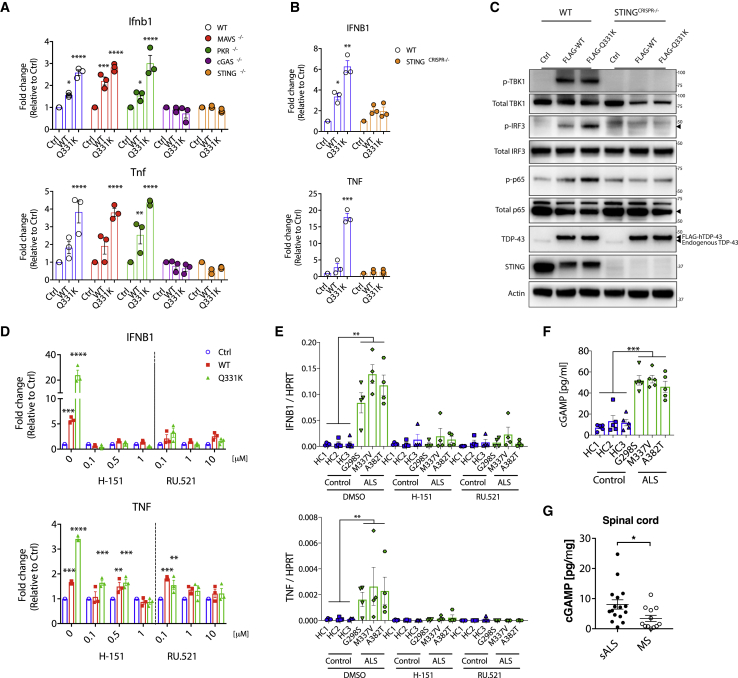
Inflammatory Signaling from TDP-43 Is Dependent on cGAS/STING (A) Vector alone (Ctrl) or plasmids encoding FLAG-tagged wild-type (WT) TDP-43 and the ALS mutation (Q331K) were transiently overexpressed in mouse embryonic fibroblasts (MEFs) genetically deficient for different innate immune sensors. Expression of *Ifnb1* and *Tnf* was measured by qPCR after 72 h and was ablated only when cGAS or STING were genetically deficient. (B) Inducible TDP-43 constructs (WT or Q331K) were transduced into WT or *STING* CRISPR knockout (KO) THP-1 cells. 72 h after Dox induction, qPCR for *IFNB1* and *TNF* was performed. (C) TDP-43-overexpressing THP-1 cells as in (B) were subjected to western blot analysis of inflammatory signaling pathways related to type I IFN and NF-κB. (D and E) The cGAS inhibitor RU.521 and STING inhibitor H-151 prevent *IFNB1* and *TNF* induction from TDP-43-overexpressing THP-1 cells used in (B) and (E) iPSC-derived motor neurons from ALS patients compared with healthy controls (RU.521, 10 μM; H-151, 1 μM). DMSO was used as a solvent control (0). (F and G) Quantification of cGAMP by ELISA from (F) cell lysates of iPSC-derived motor neurons from healthy controls and ALS patients and from (G) post-mortem spinal cord samples of patients with sporadic ALS (n = 16) or MS (n = 12). Data are mean ± SEM, pooled from 3–5 independent experiments ([A], [B], and [D]–[F]) or representative of 3 independent experiments (C). The p values were calculated using two-way ANOVA to Ctrl in (A), (B), and (D) or unpaired t test between healthy control and ALS patient iPSC-MN lines (G298S, M337V, and A382T) in (E)–(G). ^∗^p < 0.05, ^∗∗^p < 0.01, ^∗∗∗^p < 0.001, ^∗∗∗∗^p < 0.0001. See also Figure S1 and Table S1.

To examine inflammation triggered by TDP-43, we employed inducible expression of empty vector (control [Ctrl]), wild-type (WT), or ALS mutant (Q331K) TDP-43 in a mouse neuronal cell line, which confirmed upregulation of NF-κB and type I IFN pathways *in vitro* (Figures S1A–S1C). To identify the innate immune sensor regulating this response, we repeated the *in vitro* model in mouse embryonic fibroblasts (MEFs) genetically deficient for a panel of candidates that are known to regulate NF-κB and type I IFN production (Figures 1A, S1D, and S1E). Because TDP-43 is an RNA binding protein, we first interrogated sensors of cytoplasmic RNA, including RIG-I and MDA-5 (via deletion of the conserved signaling adaptor MAVS) and PKR (Figure 1A). Surprisingly, absence of these innate immune sensors did not reduce NF-κB or type I IFN activation downstream of TDP-43 overexpression. Instead, deletion of cGAS, a sensor of cytoplasmic DNA, returned activation of these pathways to baseline (Figure 1A). cGAS signals via generation of a specific cyclic dinucleotide, cGAMP, which we could also detect in response to TDP-43 (Figure S1F). cGAMP then acts to trigger STING, which, as we confirmed using genetically deficient MEFs, also prevents TDP-43-induced inflammation (Figure 1A). We then made similar findings in human myeloid THP-1 cells, in which CRISPR-mediated deletion of *STING* led to significant attenuation of type I IFN and NF-κB pathways, as demonstrated by cytokine gene expression (Figure 1B) and activation of signaling molecules via western blot (Figure 1C). Next we looked to see whether pharmacological blockade of the pathway was feasible, using recently described inhibitors of cGAS (RU.521; Vincent et al., 2017) and STING (H-151; Haag et al., 2018). Indeed, these drugs prevented expression of *IFNB1* and *TNF* in response to overexpressed WT and mutant TDP-43 (Figure 1D). We also confirmed activation of the cGAS/STING pathway in induced pluripotent stem cell (iPSC)-derived motor neurons (MNs) from ALS patients carrying familial mutations in TDP-43 (Figures 1E, 1F, and S1G–S1I). Finally, we quantified the levels of cGAMP in spinal cord samples from sporadic ALS patients and compared these with samples from cases of progressive multiple sclerosis (MS) as a neurological control (Figure 1G). This documented a significant increase in cGAMP for the ALS samples independent of age, sex, or post-mortem interval (Table S1). These results implicate cGAS as an important immune sensor regulating neuroinflammation associated with TDP-43 in ALS.

### TDP-43 Triggers mtDNA Release into the Cytoplasm

**Figure S2:**
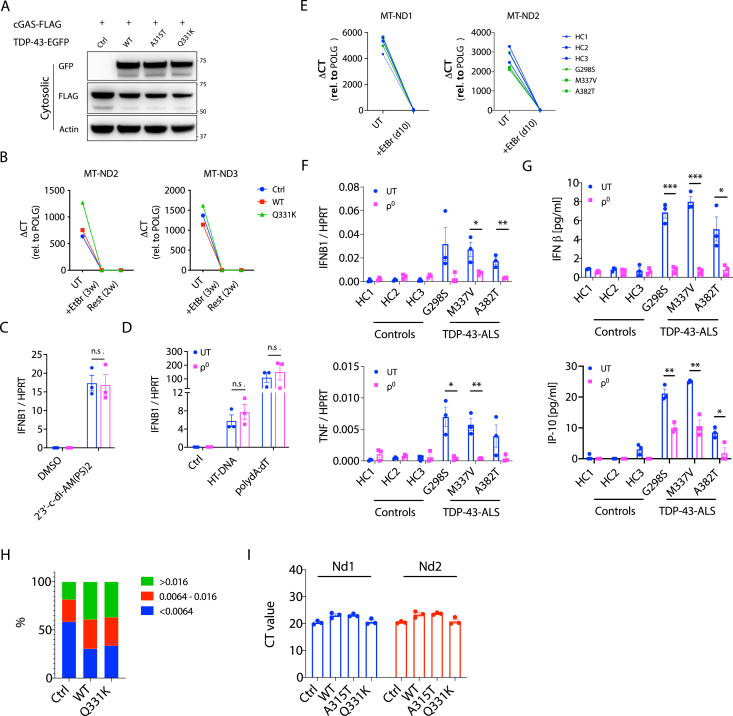
mtDNA Is a DAMP that Activates cGAS/STING Signaling, Related to Figure 2 (A) Representative western blot of FLAG-cGAS immunoprecipitation from Figure 2A. (B) Representative qPCR analysis of mitochondrial DNA (mtDNA) depletion from THP-1 cells over three weeks treatment in ethidium bromide (EtBr) and then two weeks after removing EtBr. To indicate the depletion, mitochondrial genes (*MT-ND2* and *MT-ND3*) were normalized to that of nuclear gene *POLG* as ΔCT and compared to untreated cells (UT). (C) *IFNB1* expression, measured by qPCR, in UT and ρ^0^ THP-1 cells in response to stimulation with 2′3′-c-di-AM(PS)2(Rp, Rp) (20 μM) or DMSO as solvent control for 4hrs or (D) poly(dA:dT) (1 μg/ml), HT-DNA (2 μg/ml) and equivalent Lipofectamine as control (Ctrl) for 6hrs. (E) mitochondrial genes (*MT-ND1* and *MT-ND2*) were depleted in iPSC-MNs treated with EtBr for 10 days. (F) Gene expression of *IFNB1* and *TNF*, and (G) IFNβ and IP-10 production were diminished in ρ^0^ iPSC-MNs from TDP-43-ALS patients. (H) Quantification of mtDNA nucleoid size for imaging data in Figure 2D. Nucleoid sizes were divided into three groups; < 0.0064 mm^3^, 0.0064-0.016 mm^3^ and > 0.016 mm^3^. (I) No difference in copies of mitochondrial DNA (*Nd1* and *Nd2*) was observed in total cell lysates of MEFs transfected with WT or mutant TDP-43 (A315T and Q331K). Data are mean ± SEM from 3 independent experiments. *P value*s were calculated using two-way ANOVA. ^∗^p < 0.05, ^∗∗^p < 0.01, ^∗∗∗^p < 0.001, *n.s.* not significant.

**Figure 2:**
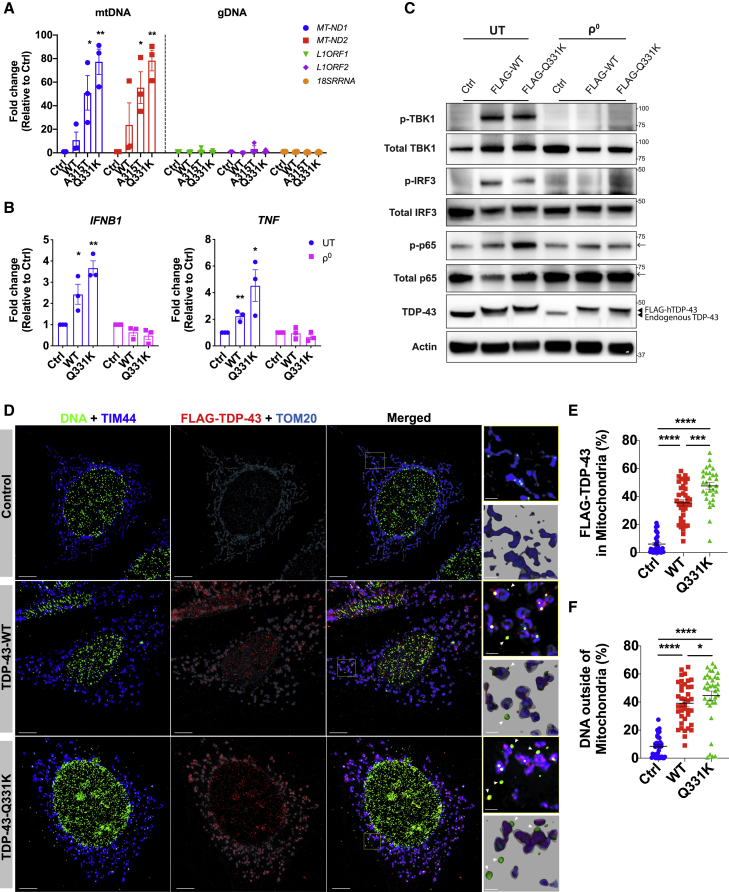
TDP-43 Causes mtDNA Release into the Cytoplasm (A) TDP-43-EGFP (WT or the ALS mutants A315T and Q331K) and FLAG-cGAS were transiently overexpressed in HEK293T cells, followed by extraction of DNA from FLAG immunoprecipitants. Direct qPCR reveals the presence of mtDNA (*MT-ND1* and *MT-ND2*) but not DNA corresponding to abundant nuclear LINE1 elements (*L1ORF1* and *L1ORF2*) or a ribosomal gene (*RNA18S*) bound to FLAG-cGAS. (B) Human THP-1 cells with inducible TDP-43 (WT or Q331K) were depleted of mtDNA using EtBr (ρ^0^). 72 h after TDP-43 induction, *IFNB1* and *TNF* expression was diminished compared with the untreated (UT) control. (C) TDP-43 overexpressing THP-1 cells as in (B) were subjected to western blot analysis of inflammatory signaling pathways related to type I IFN and NF-κB. Arrows indicate a cut in the membrane to facilitate multiple protein probing. See the Key Resources Table for uncropped blots. (D) OMX-SR microscopy reveals that TDP-43 (FLAG-tagged, red) translocates into mitochondria (TIM44, blue; TOM20, cyan) and induces relocation of DNA (anti-DNA, green) into the cytoplasm of TDP-43-overexpressing MEFs (scale bars, 5 μm). Overview images are maximum-intensity projections, and magnified images are 3D surface reconstructions using Imaris software (bottom right) (scale bars, 0.5 μm). See also Video S1. (E and F) Spatial quantification by Imaris software for (E) the percentage of FLAG-TDP-43 in mitochondria (TIM44) and (F) the percentage of DNA outside of mitochondria; 30–40 cells per group. Data are mean – SEM, pooled from 3 independent experiments ([A], [B], [E], and [F]) or representative of 3 independent experiments ([C] and [D]). The p values were calculated using two-way ANOVA to control in (A) and (B) or unpaired t test in (E) and (F). ^∗^p < 0.05, ^∗∗^p < 0.01, ^∗∗∗^p < 0.001, ^∗∗∗∗^p < 0.0001. See also Figure S2.

In sterile settings, cGAS can respond to double-stranded DNA (dsDNA) of either mitochondrial (Rongvaux et al., 2014) or nuclear origin (Ahn et al., 2014). To ascertain the source of DNA activating cGAS in response to TDP-43, we immunoprecipitated FLAG-tagged cGAS from HEK293T cells overexpressing WT or ALS mutant TDP-43 (A315T or Q331K; Figure S2A), directly followed by qPCR for the detection of mitochondrial or nuclear DNA (White et al., 2014). This showed that, in response to TDP-43 overexpression, cGAS was bound to mitochondrial DNA (mtDNA) without evidence of the most abundant nuclear DNA sequences such as LINE1 elements or the ribosomal DNA *RNA18S* (Figure 2A). It is also possible to generate cell lines deficient for mtDNA (referred to as ρ^0^) by culturing them with low-dose ethidium bromide (EtBr) (Hashiguchi and Zhang-Akiyama, 2009). mtDNA depletion from THP-1 cells containing inducible WT and mutant TDP-43 was achieved in culture with EtBr for 3 weeks, as judged by qPCR of mitochondrial gene expression (Figure S2B). We then treated the cells with doxycycline (Dox) to induce TDP-43 (WT or Q331K), followed by quantification of inflammatory cytokine expression (Figure 2B), and activation of signaling pathways downstream of cGAS/STING (Figure 2C), which returned to baseline in the ρ^0^ cells depleted of mtDNA. To ensure that EtBr treatment did not indirectly impair cGAS/STING signaling, we treated ρ^0^ cells with cGAS/STING ligands, for which responses were not reduced (Figures S2C and S2D). Of note, we also achieved significant mtDNA depletion in human iPSC-MNs over a shorter course of 10 days of EtBr treatment (Figure S2E), after which cytokine expression and IFN production were consistently decreased (Figures S2F and S2G). Taking an independent approach, we imaged cells and observed leakage of mtDNA into the cytoplasm in response to overexpression of WT TDP-43, which was further augmented in cells with the ALS-associated mutant TDP-43 (Figures 2D–2F; Video S1). Consistent with earlier reports regarding mtDNA stress (West et al., 2015), nucleoid size was increased because of TDP-43 (Figure S2H); however, TFAM levels appear normal, and mtDNA copy numbers were not increased (Figures S1I and S2I). Therefore, we identified mtDNA leakage into the cytosol as the trigger for cGAS/STING activation because of TDP-43 and next queried mechanisms for TDP-43 entry into and mtDNA release from mitochondria.

Video S1. TDP-43 Triggers mtDNA Release into the Cytoplasm, Related to Figure 2 Control MEFs or those overexpressing TDP-43 (WT or Q331K) were stained for TDP-43 (FLAG-tagged, red), mitochondrial inner membrane (TIM44, blue), mitochondrial outer membrane (TOM20, gray) and DNA (anti-DNA, green) then imaged by OMX-SR microscopy. This video demonstrates mtDNA release into the cytoplasm in response to TDP-43 proteinopathy.

### TDP-43 Entry into Mitochondria Requires the Translocase Subunit AGK

**Figure 3:**
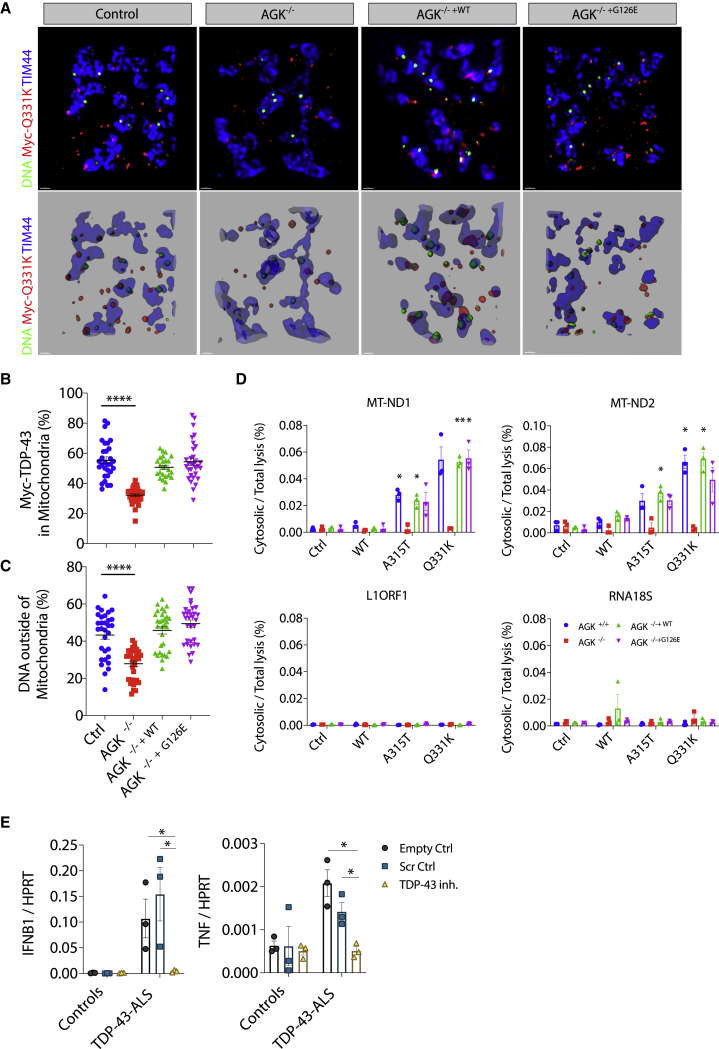
TDP-43 Entry into Mitochondria Requires AGK Independent of Its Lipid Kinase Function (A) OMX-SR microscopy reveals that import of TDP-43 (Myc-tagged, red) into mitochondria (TIM44, blue) and TDP-43-induced relocation of DNA (anti-DNA, green) into the cytoplasm are ablated in HEK293T cells lacking the TIM22 regulatory subunit AGK (scale bars, 0.5 μm). Overview images are maximum-intensity projections (top) or 3D surface reconstructions using Imaris software (bottom). See also Video S2. (B and C) Spatial quantification by Imaris software for (B) the percentage of Myc-TDP-43 in mitochondria (TIM44) and (C) the percentage of DNA outside of mitochondria in control, *AGK*^*−/−*^, *AGK*^*−/− +WT*^, or *AGK*^*−/− +G126E*^ HEK293T cells; 30 cells per group. (D) TDP-43-induced (WT, mutant A315T and Q331K) mtDNA release (cytosolic/total lysis, percent) is ablated in cells that lack AGK. (E) Treatment with the TDP-43 inhibitor peptide (PM1; 1 μM for 24 h) prevents induction of *IFNB1* and *TNF* in TDP-43-ALS patient iPSC-MNs. Data are mean ± SEM from 3 independent experiments. The p values were calculated using one-way or two-way ANOVA to Ctrl in (B)–(D) or unpaired t test between healthy Ctrl and ALS patient iPSC-MNs in (E). ^∗^p < 0.05, ^∗∗^p < 0.01, ^∗∗∗∗^p < 0.0001. See also Figure S3.

**Figure S3:**
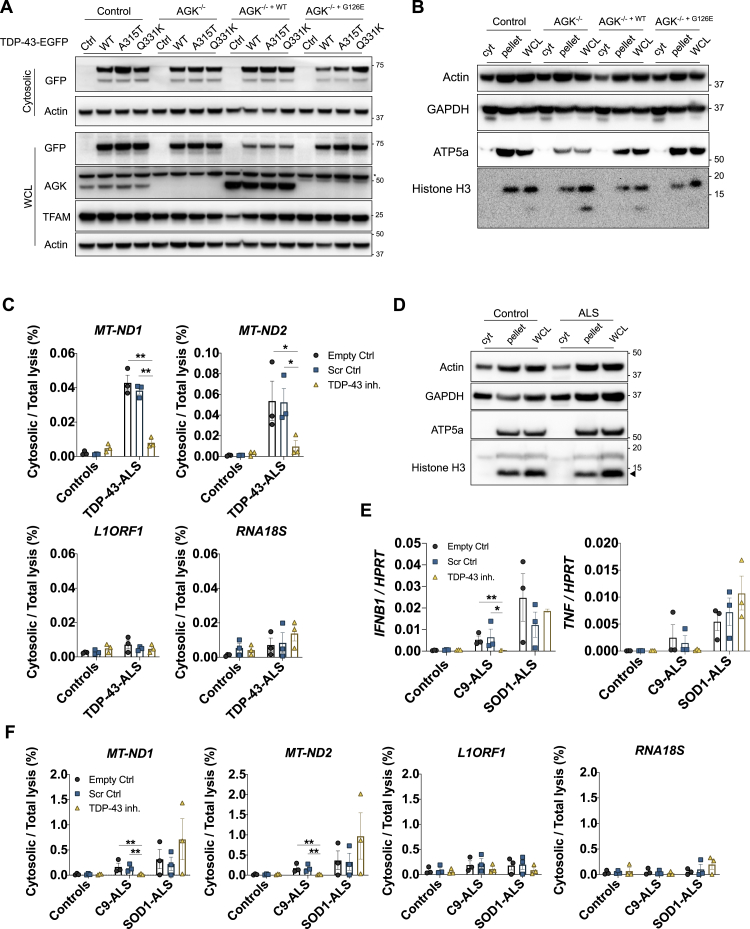
mtDNA Leakage Is Dependent on TDP-43 Entry into Mitochondria, Related to Figure 3 (A) Representative western blot analysis of GFP-tagged TDP-43 (WT, A315T and Q331K), AGK, TFAM and Actin in Flp-In control, *AGK*^*−/−*^, *AGK*^*−/− +WT*^ or *AGK*^*−/− +G126E*^ HEK293T cells. (B) Representative western blot analysis of cells in panel (A), to establish the purity of Digitonin lysis cytosolic fraction (cyt) compared to the pellet and RIPA whole cell lysate (WCL), using the subcellular markers indicated. (C) iPSC-MNs from healthy controls (HC1, HC2, HC3) and TDP-43-ALS patients (G298S, M337V, A382T) were treated with 1 μM of control peptide (Scr ctrl) or inhibitor peptide (PM1) for 24h and were lysed in 0.0045% Digitonin buffer for cytosolic fraction or 1x RIPA buffer for whole cell lysate control (WCL) after which DNA was extracted and directly amplified by qPCR to reveal the reduced presence of mtDNA (*MT-ND1* and *MT-ND2*) in the cytoplasm. LINE1 element (*L1ORF1*) and a ribosomal gene (*RNA18S*) were not affected. (D) Representative western blot analysis of control and ALS patient iPSC-MNs, to establish the purity of Digitonin lysis cytosolic fraction (cyt) compared to the pellet and RIPA whole cell lysate (WCL), using the subcellular markers indicated. (E) iPSC-MNs from healthy controls (HC4, HC5, HC6), C9-ALS patients and SOD1-ALS patients were analyzed by qPCR for expression of *IFNB1* and *TNF*, or (F) subjected to subcellular fractionation and DNA quantification as in panel (C). Data are mean ± SEM from 3 independent experiments with three controls or patients per group. *P value*s were calculated using two-way ANOVA to Empty control (Ctrl) per genotype. ^∗^p < 0.05, ^∗∗^p < 0.01.

Several studies now demonstrate the presence of TDP-43 in mitochondria using model systems and in ALS patient neurons post mortem (Davis et al., 2018; Ruan et al., 2017; Salvatori et al., 2018; Wang et al., 2019). Additionally, it has been reported that TDP-43 gains access to the mitochondrial matrix via the mitochondrial import inner membrane translocase TIM22 (Wang et al., 2016). We confirmed this and found that TDP-43 (WT or Q331K) did not release mtDNA in cells deficient for the TIM22 subunit *AGK* (Kang et al., 2017; Figures 3A–3D, S3A, and S3B; Video S2). However, AGK has an additional function independent of the TIM22 translocase machinery, acting as a lipid kinase (Kang et al., 2017; Vukotic et al., 2017). To ensure that the effect of TIM22 deletion was not an indirect effect of deficient lipid kinase activity, cells were reconstituted with mutant AGK(G126E), which cannot phosphorylate lipids. Similar to WT AGK, this mutant AGK, which lacks lipid kinase activity, still promoted mitochondrial localization of TDP-43 and the release of mtDNA (Figures 3A–3D). TIM22 normally functions to import multi-pass transmembrane proteins, which TDP-43 is not; however, it does possess several stretches of hydrophobic amino acids that in part resemble transmembrane domains (Wang et al., 2016). Some of these hydrophobic motifs have been identified to be critical for the mitochondrial import of TDP-43 and lead to development of competitive peptide inhibitors based on the motif itself, fused to a viral TAT peptide to facilitate delivery into the cell. These competitive peptide inhibitors prevent the mitochondrial import of TDP-43 (Wang et al., 2016), and we show that these inhibitors also prevent subsequent leakage of mtDNA into the cytosol of ALS patient iPSC-derived motor neurons (Figures S3C and S3D). Furthermore, these inhibitors are reported to have therapeutic benefits in mouse models of ALS driven by TDP-43 (Wang et al., 2016), and that is consistent with their anti-inflammatory effect when we examined expression of *IFNB1* and *TNF* in ALS patient iPSC-derived motor neurons (Figure 3E). As a comparison, we obtained iPSCs from ALS patients with repeat expansions in C9orf72 (C9-ALS), who are also known to develop TDP-43 pathology (DeJesus-Hernandez et al., 2011), and with mutations in SOD1 (SOD1-ALS), which results in mitochondrial damage without any evidence of TDP-43 pathology (Mackenzie et al., 2007; Tan et al., 2007). Notably, the competitive TDP-43 inhibitors prevent *IFNB1* and *TNF* expression and mtDNA leakage into the cytosol for C9orf72 but not SOD1 iPSC-derived motor neurons (Figures S3E and S3F). Together, these data document that mtDNA release into the cytosol and subsequent *IFNB1* expression depends on hydrophobic motifs in TDP-43 and mitochondrial import regulated by AGK.

Video S2. TDP-43 Import into Mitochondria Dependent on AGK, Related to Figure 3 HEK293T Flp-In control, *AGK*^*−/−*^, *AGK*^*−/− +WT*^ or *AGK*^*−/− +G126E*^ cells expressing TDP-43 (Q331K) were stained for TDP-43 (FLAG-tagged, red), mitochondrial inner membrane (TIM44, blue) and DNA (anti-DNA, green) then imaged by OMX-SR microscopy. This video demonstrates that TDP-43 entry into mitochondria requires AGK, independent of its lipid kinase function.

### TDP-43 Triggers mtDNA Release into the Cytoplasm via the mPTP

**Figure S4:**
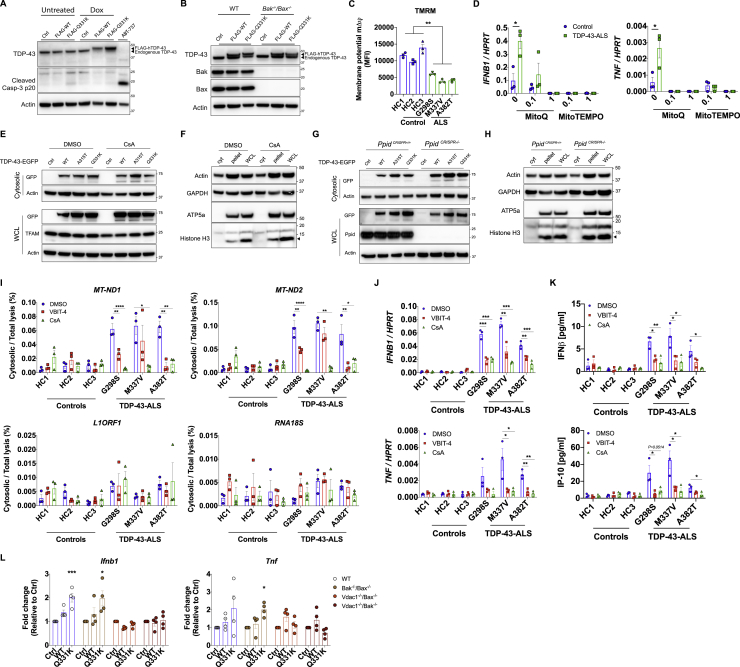
TDP-43 Releases mtDNA via the mPTP, Related to Figure 4 (A) Representative western blot of cleaved caspase-3 in TDP-43-overexpressing *Mcl1*^−/−^ MEFs 72hrs post doxycycline (Dox) treatment, or treated with ABT-737 to induce apoptosis (t = 4h). (B) Representative western blot of Bak, Bax, TDP-43 and actin from cells in Figure 4A. (C) iPSC-derived MNs from healthy controls and ALS patients carrying mutations in TDP-43 were stained with Tetramethylrhodamine Methyl Ester (TMRM) to probe mitochondrial membrane potential (mΔΨ) and quantified by FACS analysis (MFI: mean fluorescence intensity). (D) Mitochondrial ROS inhibitors, mitoQ and mitoTEMPO (0.1-1 μM), potently prevent induction of *IFNB1* and *TNF* in TDP-43-ALS iPSC-MNs. (E-F) Representative western blot analysis of cytosolic lysates (0.025% digitonin) and WCL (1x RIPA) from HEK293T cells treated with CsA (12.5 μM) or from (G-H) CRISPR *Ppid* knockout MEFs transfected with TDP-43-EGFP (WT, A315T and Q331K, 2.5 μg) or untransfected (Ctrl). Cytosolic fraction purity was confirmed using the subcellular markers indicated (I) Treatment with VBIT-4 and CsA (10uM, t = 24h) reduces mtDNA (*MT-ND1* and *MT-ND2*) release into the cytoplasm as performed in Figure S3C, (J) reduces expression of *IFNB1* and *TNF* as determined by qPCR, and (K) reduces production of IFNβ and IP-10 quantified by ELISA. (L) CRISPR *Vdac1* knockout MEFs display no TDP-43-induced *Ifnb1* and *Tnf* expression. Data are mean ± SEM from 3-4 independent experiments. *P value*s were calculated using un-paired t test between healthy control and TDP-43-ALS patient iPSC-MN lines or one-way ANOVA to DMSO or vector control. ^∗^p < 0.05, ^∗∗^p < 0.01, ^∗∗∗^p < 0.001, ^∗∗∗∗^p < 0.0001.

**Figure 4:**
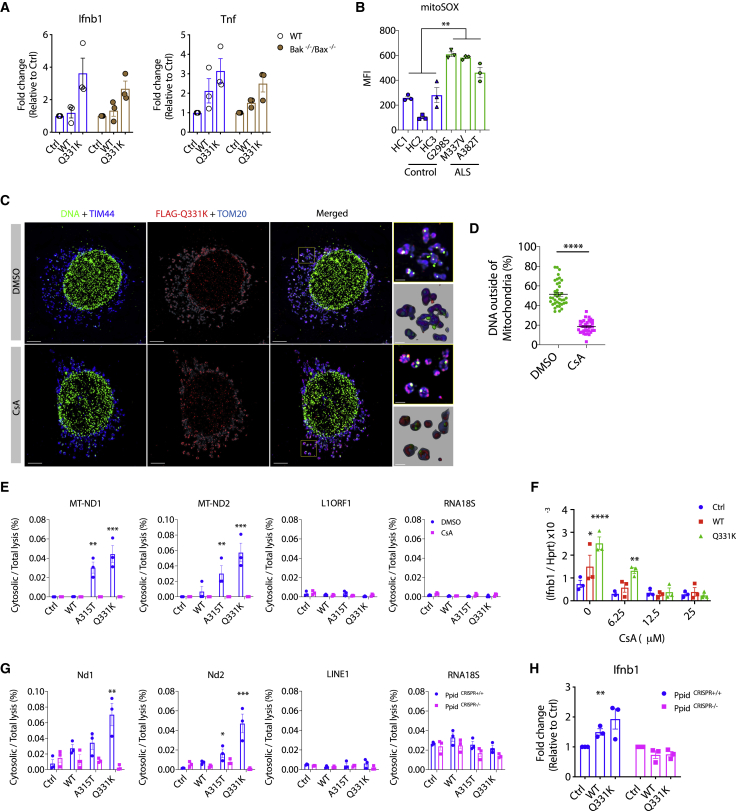
TDP-43 Causes mtDNA Release to the Cytoplasm via the mPTP (A) Plasmids encoding TDP-43 (WT or Q331K) were transiently overexpressed in MEFs that are genetically deficient for Bax and Bak. Expression of *Ifnb1* and *Tnf* was measured by qPCR after 72 h. (B) Human iPSC-derived motor neurons from healthy controls and ALS patients carrying mutations in TDP-43 (G298S, M337V, and A382T) were treated with mitoSOX red to quantify mitochondrial ROS 2 weeks after terminal differentiation and then subjected to fluorescence-activated cell sorting (FACS) analysis (MFI, mean fluorescence intensity). (C) OMX-SR microscopy reveals that TDP-43-induced (FLAG-tagged, red) relocation of DNA (anti-DNA, green) from mitochondria (TIM44, blue; TOM20, cyan) into the cytoplasm was reduced significantly by inhibition of the mPTP (CsA, 12.5 μM) in TDP-43 mutant (Q331K)-overexpressing MEFs (scale bars, 5 μm). DMSO was used as a solvent control. Overview images are maximum-intensity projections, and magnified images are 3D surface reconstructions using Imaris software (bottom right) (scale bars, 0.5 μm). See also Video S3. (D) Spatial quantification by Imaris software for the percentage of DNA outside of mitochondria (TIM44); 30–40 cells per group. (E and F) Inhibition of the mPTP (CsA, 12.5 μM) in HEK293T cells prevents mtDNA cytosolic accumulation (cytosolic/total lysis, percent) and (F) prevents *Ifnb1* gene expression relative to *Hprt*, as induced by TDP-43 transient overexpression (WT, A315T, or Q331K). (G and H) CRISPR-mediated genetic deletion of the mPTP component *Ppid* also abolished mtDNA release into the cytoplasm and (H) downstream *Ifnb1* gene expression. Data are mean ± SEM, pooled from 3 independent experiments ([A], [B], and [D]–[H]) or representative of 3 independent experiments (C). The p values were calculated using unpaired t test in (B) and (D), two-way ANOVA to control in (A) and (E)–(H). ^∗^p < 0.05, ^∗∗^p < 0.01, ^∗∗∗^p < 0.001, ^∗∗∗∗^p < 0.0001. See also Figure S4.

After confirming the mode of entry of TDP-43 into mitochondria, we sought to identify the mechanism for mtDNA release. It has been reported that TDP-43 is capable of inducing apoptosis under certain conditions, which could trigger Bak/Bax permeabilization of the outer mitochondrial membrane and leakage of DNA into the cytoplasm (McArthur et al., 2018). However, in these assays, we did not see evidence of apoptosis, as indicated by cleaved caspase-3 (Figure S4A), and furthermore, the deletion of Bak/Bax had no effect on inflammatory cytokine gene expression in response to TDP-43 (WT or Q331K) (Figures 4A and S4B). Instead, we observed hallmarks of mitochondrial destabilization, such as upregulation of ROS (Figure 4B) and loss of membrane potential (mΔψ) (Figure S4C) in ALS patient iPSC-derived motor neurons. Additionally, inhibiting mitochondrial ROS prevents inflammation in response to TDP-43 (Figure S4D). These observations potentially suggest destabilization and, thus, opening of the mitochondrial permeability transition pore (mPTP) (Du et al., 2008; Nguyen et al., 2011), which can lead to mtDNA release (García and Chávez, 2007). In agreement, pharmacological inactivation of the mPTP using cyclosporin A (CsA) prevented TDP-43(Q331K)-mediated mtDNA leakage into the cytoplasm (Figures 4C–4E, S4E, and S4F; Video S3), thus attenuating *IFNB1* expression (Figure 4F). This was also observed in patient iPSC-derived MNs (Figures S4I–S4K). Similarly, genetic deletion of *Ppid* (encoding the mPTP component PPID, also referred to as Cyclophilin D) ameliorated TDP-43-mediated mtDNA release into the cytoplasm (Figures 4G, S4G, and S4H) and downstream inflammation (Figure 4H). Another mitochondrial component that may participate in release of mtDNA fragments because of oxidative stress is voltage-dependent anion channel 1 (VDAC1) (Kim et al., 2019). In agreement with this, we observed that the VDAC1 oligomerization inhibitor VBIT-4 prevents cytosolic accumulation of mtDNA and inflammation in ALS patient iPSC-derived motor neurons (Figures S4I–S4K). We also confirmed that genetic deletion of VDAC1 in MEFs prevents *Ifnb1* and *Tnf* expression driven by overexpressed TDP-43 (Figure S4L). These MEF cell lines were obtained from a previous study (Chin et al., 2018) and are coincidently deficient in either Bak or Bax, but again, we confirmed that those genes are not involved in TDP-43-dependent mtDNA release (Figure S4L). These *in vitro* data demonstrate a mechanism by which TDP-43 can mislocalize into mitochondria, opening the mPTP and resulting in VDAC1-dependent mtDNA leakage into the cytoplasm.

Video S3. Inhibition of mPTP Prevents mtDNA Release due to TDP-43, Related to Figure 4 TDP-43-Q331K expressing MEFs were treated with CsA (12.5 μM) or DMSO as control, and stained for TDP-43 (FLAG-tagged, red), mitochondrial inner membrane (TIM44, blue), mitochondrial outer membrane (TOM20, gray) and DNA (anti-DNA, green), then imaged by OMX-SR microscopy. This video demonstrates that TDP-43-induced relocation of DNA from mitochondria into the cytoplasm was significantly reduced by inhibition of the mPTP.

### Genetic Deletion of *Sting* Mitigates Disease in an ALS Mouse Model

**Figure S5:**
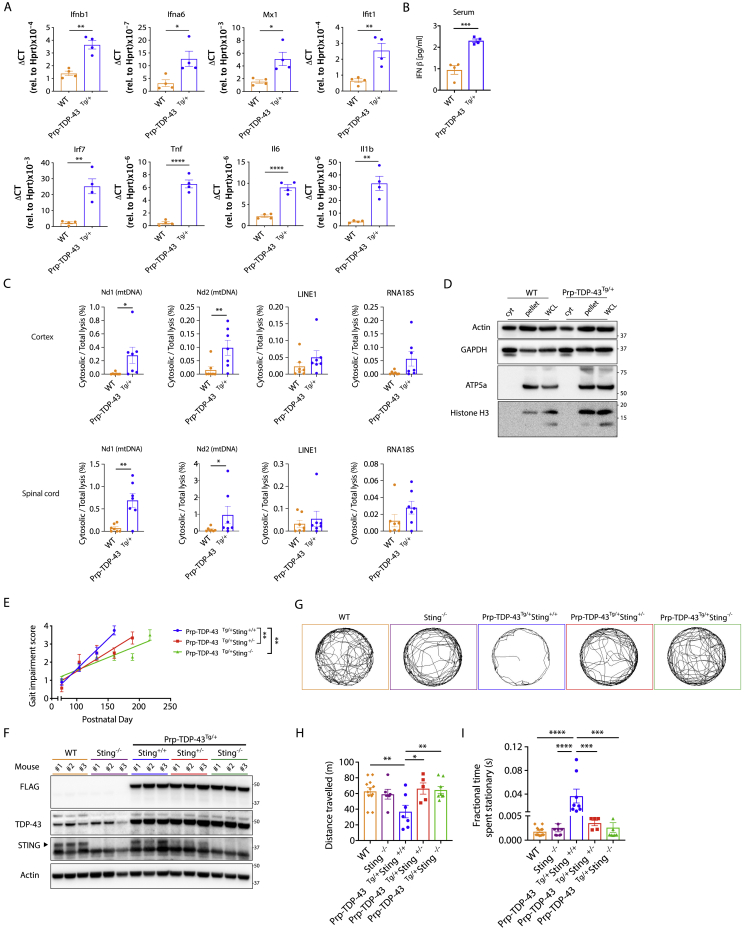
Disease Incidence and Progression in Prp-TDP-43^Tg/+^ Mice, Related to Figure 5 (A) qPCR analysis of type I IFNs (*Ifnb1* and *Ifna6*), interferon-stimulated genes (*Mx1*, *Ifit1* and *Irf7*) and NF-κB genes (*Tnf*, *Il6* and *Il1b*) are presented for WT and Prp-TDP-43^Tg/+^ bone marrow derived macrophages, taken from mice at 150 days of age. The mRNA expression was normalized to *Hprt* as relative gene expression (mean ± SD from 4 mice per group). (B) Serum IFNβ from WT and Prp-TDP-43^Tg/+^ mice was quantified by ELISA at day 150. (C) Single cell suspensions of cortex and spinal cord from WT and Prp-TDP-43^Tg/+^ mice (n = 7, age 120 days) were subjected to subcellular fractionation followed by direct qPCR for mtDNA (*Nd1* and *Nd2*), *LINE1* element and ribosome control (*RNA18S*). (D) Representative western blot analysis of single cell suspensions from cortex and spinal cord of WT and Prp-TDP-43^Tg/+^ mice, to establish the purity of Digitonin lysis cytosolic fraction (cyt) compared to the pellet and RIPA whole cell lysate (WCL), using the subcellular markers indicated (E) Genetic deletion of *Sting* significantly mitigates the rate of disease progression after onset (slope of linear regression for gait impairment across lifespan). Data are mean ± SEM with *P value*s 0.0018 in Prp-TDP-43^Tg/+^*Sting*^+/−^ and 0.0015 in Prp-TDP-43^Tg/+^*Sting*^−/−^ when compared to Prp-TDP-43^Tg/+^*Sting*^*+/+*^. (F) Representative western blot of transgenic FLAG-TDP-43 mutant (A315T), TDP-43, STING and Actin in brain lysates of Prp-TDP-43^Tg/+^ strains and control strains used in Figure 5. Three mice analyzed per group at 150 days of age. (G) Video captured from the OF test was analyzed using ImageJ and MouseMove then presented as representative cumulative trajectories of male WT (n = 12), *Sting*^−/−^ (n = 6), Prp-TDP-43^Tg/+^*Sting*^+/+^ (n = 8), Prp-TDP-43^Tg/+^*Sting*^+/−^ (n = 5) and Prp-TDP-43^Tg/+^*Sting*^−/−^ (n = 8) mice at 130 days. (H) Quantification of test data shows that heterozygous and homozygous deletion of *Sting* significantly restores locomotor activity in Prp-TDP-43^Tg/+^ models of ALS relative to WT controls (n = 12) in terms of distance traveled and (I) their fractional time spent stationary during 10 min OF test at 120-130 days. Animals studied here were all males. Data are mean ± SEM from 4 independent neurological behavior tests. *P value*s were calculated using un-paired t test between two groups (A, B, C) or one-way ANOVA (E, H, I) to Prp-TDP-43^Tg/+^*Sting*^*+/+*^. ^∗^p < 0.05, ^∗∗^p < 0.01, ^∗∗∗^p < 0.001, ^∗∗∗∗^p < 0.0001.

**Figure 5:**
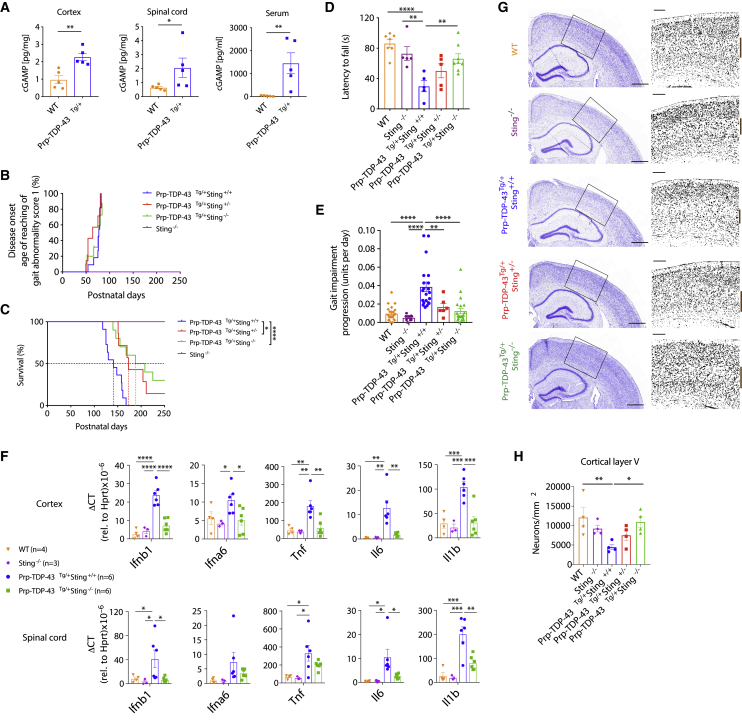
Genetic Deletion of *Sting* Mitigates Disease in an ALS Mouse Model (A) Quantification of cGAMP in the cortex, spinal cord, and serum of WT mice and mice that are transgenic for the human TDP-43 mutant allele A315T (Prp-TDP-43^Tg/+^) (n = 5) at the experimental endpoint. See animal phenotype scoring. (B) Genetic deletion of *Sting* does not change disease onset in Prp-TDP-43^Tg/+^ mice (disease incidence on the day when gait impairment first achieves a score of 1). (C) Prp-TDP-43^Tg/+^ mice (n = 11) develop progressive neurodegenerative disease that requires euthanasia at a median of 148 days. Heterozygous (n = 9) or homozygous (n = 10) loss of *Sting* significantly increases the lifespan. (D and E) At 120 days, Prp-TDP-43^Tg/+^ mice exhibit significantly decreased latency to fall in a rotarod test (n = 5–8) and (E) significant gait impairment (n = 6–21), which are greatly rectified by genetic deletion of *Sting*. (F) qPCR of inflammatory gene expression relative to *Hprt* in the cortex and spinal cord reveals that increased levels of type I IFN- and NF-κB-dependent cytokines are greatly reduced because of genetic deletion of *Sting* (n = 3–6). (G) Representative Nissl body staining (cresyl violet) of a coronal section (scale bars, 5 mm) through the brain of WT and Prp-TDP-43^Tg/+^ mice with and without the genetic deletion of *Sting* at 150 days of age. Overview images are selected magnified grayscale images (scale bars, 200 μm). (H) Quantification of cortical layer V neurons marked by a brown bar in (G) (n = 4). All animals studied here were males. Data are mean ± SEM. The p values were calculated using unpaired t test between two groups in (A) or one-way ANOVA to Prp-TDP-43^Tg/+^*Sting*^+/+^ in (B)–(F) and (H). ^∗^p < 0.05, ^∗∗^p < 0.01, ^∗∗∗^p < 0.001, ^∗∗∗∗^p < 0.0001. See also Figure S5.

To establish whether the cGAS/STING pathway is responsible for neuroinflammation in response to aberrant TDP-43 *in vivo*, we used the well-described murine model of ALS with human TDP-43 (p.A315T) overexpression in mice (referred to as Prp-TDP-43^Tg/+^) (Wegorzewska et al., 2009). When given access to a jellified diet, this strain avoids early lethality because of gastrointestinal blockage and succumbs to symptoms of motor neuron degeneration at a median age of 148 days for males. We were also able to observe induction of inflammatory cytokines in bone marrow-derived macrophages, and IFNβ in the serum of mice with established disease (Figures S5A and S5B). More specifically, we were able to observe elevated levels of the cGAS signaling molecule cGAMP in the spinal cord and cortex of these mice on autopsy and also in the circulation of mice with established disease (Figure 5A). In agreement with the activation of cGAS/STING, we detected mtDNA in cytosolic lysates prepared from single-cell suspensions of Prp-TDP-43^Tg/+^ mouse cortices or spinal cords (Figures S5C and S5D). Following this, we crossed the Prp-TDP-43^Tg/+^ strain to *Sting*-deficient mice. Although we observed no difference in early disease onset, as determined by motor impairment (Figure 5B), progression of disease was slowed by 58% in Prp-TDP-43^Tg/+^ *Sting*^−/−^ mice (Figure S5E). Overall, this leads to a significant extension of the lifespan by 40% to a median of 208 days (Figure 5C) without decreasing the expression of TDP-43 (Figure S5F). Notably, deletion of only a single allele of *Sting* also afforded significant protection, with disease progression slowed by 37% (Figure S5E), and survival increased to a median of 173 days (Figure 5C). For patients with neurodegeneration associated with TDP-43, it would be essential to ameliorate disease symptoms. On day 120, we could observe that TDP-43 mutant mice were unable to maintain latency in the gold-standard “rotarod” test and suffered progressive deterioration of gait; however, mice deficient for *Sting* performed significantly better (Figures 5D and 5E). At this time point, deletion of *Sting* also significantly increased the distance traveled by TDP-43 mutant mice in an open field test and reduced their fractional time spent stationary (Figures S5G–S5I). Finally, we confirmed that the beneficial effect of deleting *Sting* in the Prp-TDP-43^Tg/+^ model was associated with decreased neuroinflammation and neurodegeneration. Specifically, inflammatory type I IFN and NF-κB gene expression was no longer upregulated in the cortex and spinal cord (Figure 5F), and there was no longer a significant loss of neurons from cortical layer V, as quantified by Nissl body staining (Figures 5G and 5H). Therefore, loss of *Sting* results in a dramatic reduction in disease progression for an aggressive ALS-associated TDP-43 mutation in mice. The observation that deletion of only one allele of *Sting* also attenuates disease suggests that pharmacological inhibition of cGAS/STING could be clinically efficacious.

### STING Inhibition Ameliorates Neurodegeneration *In Vitro* and *In Vivo*

**Figure 6:**
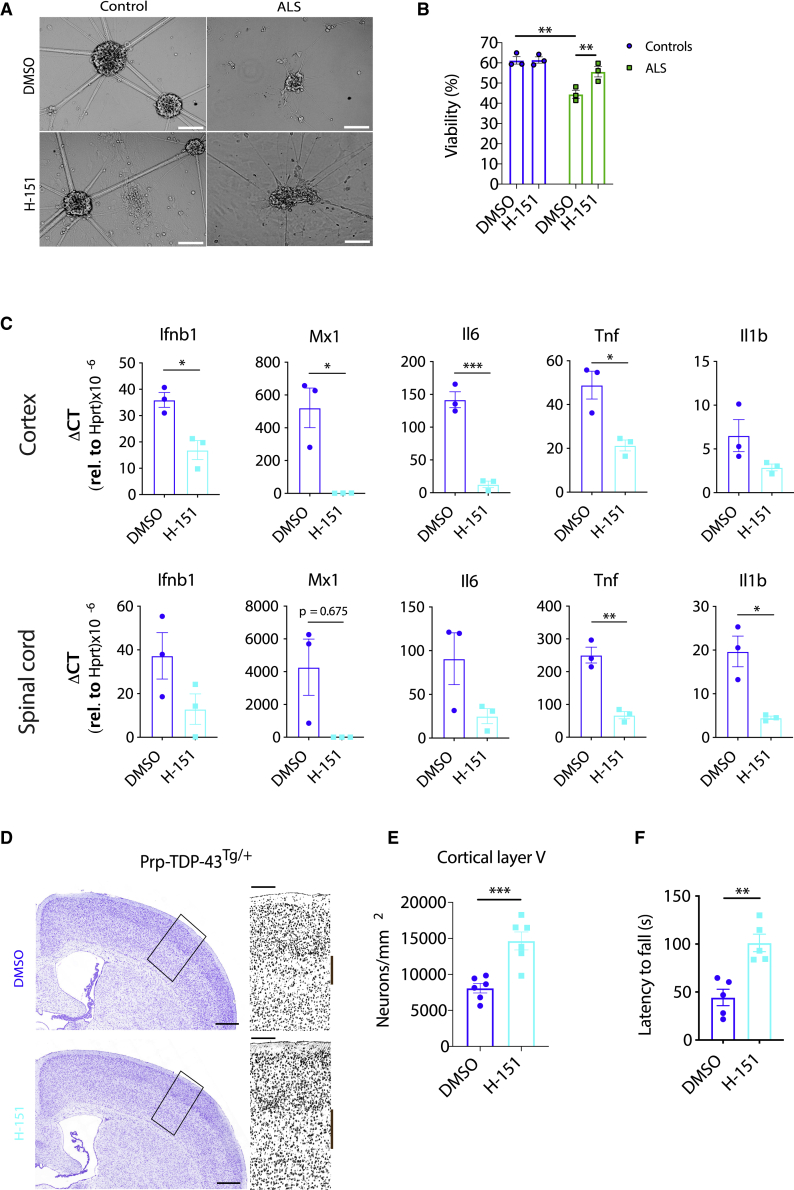
STING Inhibition Ameliorates Neurodegeneration *In Vitro* and *In Vivo* (A) Representative bright-field images demonstrate that H-151 (1 μM) prevents the death of iPSC-derived motor neurons from TDP-43-ALS patients 28 days after terminal differentiation (scale bars, 50 μm). (B) Quantification of cell death in (A), as measured by LDH release assay. (C) Prp-TDP-43^Tg/+^ mice were injected intraperitoneally (i.p.) with H-151 (210 μg) three times per week for 4 weeks, starting at disease onset at day 110 of age. This treatment significantly diminishes proinflammatory cytokine gene expression in the cortex and spinal cord, as seen by qPCR (n = 3). (D) Neuron loss was imaged by cresyl violet staining of a coronal section (scale bars, 5 mm). A representative image is shown, with selected magnified grayscale images highlighting cortical layer V neurons marked by a brown bar (scale bars, 200 μm). (E) Automated quantification of cortical layer V neurons from (D) (n = 6). (F) H-151-treated mice demonstrated improved performance in the rotarod test compared with DMSO-treated mice (n = 5). All animals studied here were males. Data are mean ± SEM. The p values were calculated using two-way ANOVA in (B) or unpaired t test between two groups in (C), (E), and (F). ^∗^p < 0.05, ^∗∗^p < 0.01, ^∗∗∗^p < 0.001.

Interestingly, we observed that ALS patient iPSC-derived motor neurons had impaired survival 28 days after terminal differentiation, whereas control motor neurons were still predominantly viable (Figures 6A and 6B). Addition of the STING inhibitor H-151 during the final 28 days of culture prevented excess motor neuron death, suggesting a cell-intrinsic role of the cGAS/STING pathway (Figures 6A and 6B). Based on these results, we proceeded to test the STING inhibitor *in vivo* for the Prp-TDP-43^Tg/+^ mouse model of ALS. Because we demonstrated that genetic deletion of *Sting* did not alter the onset of disease, we initiated treatment when symptoms were first observed on day 110. Following 28 days of treatment, we examined the expression of type I IFN and NF-κB genes, which were decreased significantly in the cortex and spinal cord (Figure 6C). We also quantified neurons in cortical layer V, whose loss was prevented by H-151 treatment (Figures 6D and 6E). These protective effects significantly ameliorate disease progression, as demonstrated by better performance in the rotarod test (Figure 6F). This administration regimen of a STING inhibitor at disease initiation provides hope that early intervention in patients could also provide a significant delay in neurodegeneration and highlights the translational potential of targeting this pathway in ALS.

## Discussion

Inflammation is gaining increased recognition for its role in neurodegeneration, and the cGAS/STING pathway is now specifically implicated in conditions such as Parkinson’s disease (Sliter et al., 2018). STING can drive activation of NF-κB and type I IFN pathways, both of which are elevated in ALS and could contribute to progression of TDP-43-driven neurodegeneration. Our analysis of patient iPSC-derived motor neuron survival *in vitro* suggests that the role of STING is cell intrinsic; however, it is possible that there are additional roles of other cell types in the CNS *in vivo*. Studies with cell-type-specific deletion of STING or IFNAR in mouse models of ALS would help to elucidate this further. Interestingly, the adaptor protein downstream of STING, TBK1, is mutated and haploinsufficient in familial ALS (Cirulli et al., 2015; Freischmidt et al., 2015). Indeed, our data suggest that some TBK1 signaling must remain intact to initiate TDP-43-driven inflammation via cGAS/STING, although it may be that partial loss of TBK1 can ameliorate end-stage disease progression in a SOD1 mouse model (Brenner et al., 2019). In contrast, total deletion of TBK1 appears to trigger an entirely different pathway of RIPK1-dependent necroptosis, resulting in lethality (Xu et al., 2018).

TDP-43 entry into mitochondria appears to be highly conserved evolutionarily, with evidence of this in yeast (Ruan et al., 2017) and demonstrated for mice *in vivo* (Davis et al., 2018)and in patients with ALS (Wang et al., 2019). However, in some contexts, mitochondrial localization of TDP-43 has not been observed (Arnold et al., 2013; Hatzipetros et al., 2014; White et al., 2018). Generally, it appears that mouse models with lower levels of TDP-43 expression provide less evidence of cytoplasmic or mitochondrial TDP-43 localization, in agreement with less pronounced neurological phenotypes. The mouse model we employed represents the severe end of the phenotypic spectrum, associated with early lethality because of neurodegeneration in the myenteric plexus of the colon, leading to gastrointestinal dysfunction and death around 100 days of age unless the mice are fed a jellified diet (Herdewyn et al., 2014). On this diet, it has been observed that mice survive to develop the key hallmarks of neurodegeneration (Coughlan et al., 2016), including mitochondrial localization of TDP-43 (Wang et al., 2019). Our *in vitro* data suggest that this leads to increased mtROS, associated with opening of the mPTP and leakage of mtDNA in a VDAC1-dependent process that is independent of Bak/Bax. Moreover, our results confirm that endogenous levels of TDP-43 drive cytoplasmic accumulation of mtDNA and cGAS/STING activation in ALS patient iPSC-derived motor neurons and spinal cord samples.

This work identifies a novel mechanism by which neuroinflammation is triggered in TDP-43 proteinopathies. Our assessment of cells lines, a mutant TDP-43 mouse model, and human ALS-affected spinal cord samples provides support for a model where TDP-43 liberates mtDNA into the cytoplasm via the mPTP to activate cGAS/STING signaling. Given that a range of cGAS and STING inhibitors have been developed recently (Haag et al., 2018; Vincent et al., 2017), including those which we have shown here to prevent TDP-43-induced inflammation, it should be possible to determine whether targeting this pathway can improve the symptoms of neuronal decline in patients with disease involving TDP-43 proteinopathy.

## STAR★Methods

### Key Resources Table

**Table:** 

REAGENT or RESOURCE	SOURCE	IDENTIFIER
**Antibodies**		

Rabbit monoclonal anti-phospho-TBK1/NAK (Ser172) (D52C2) antibody	Cell Signaling Technology	Cat# 5483; RRID: AB_10693472
Rabbit monoclonal anti-TBK1/NAK (D1B4) antibody	Cell Signaling Technology	Cat# 3504; RRID: AB_2255663
Rabbit monoclonal anti-phospho-IRF-3 (Ser396) (D6O1M) antibody	Cell Signaling Technology	Cat# 29047; RRID: AB_2773013
Rabbit polyclonal anti-IRF-3 (FL-425) antibody	Santa Cruz Biotechnology	Cat# sc-9082; RRID: AB_2264929
Rabbit monoclonal anti-phospho-NF-κB p65 (Ser536) (93H1) antibody	Cell Signaling Technology	Cat# 3033; RRID: AB_331284
Rabbit monoclonal anti-NF-κB p65 (C22B4) antibody	Cell Signaling Technology	Cat# 4764; RRID: AB_823578
Rabbit monoclonal anti-TFAM (D5C8) antibody	Cell Signaling Technology	Cat# 8076; RRID: AB_10949110
Rabbit monoclonal anti-STING (D2P2F) antibody	Cell Signaling Technology	Cat# 13647; RRID: AB_2732796
Rabbit monoclonal anti-cleaved Caspase-3 (Asp175) (5A1E) antibody	Cell Signaling Technology	Cat# 9664; RRID: AB_2070042
Rabbit polyclonal anti-GFP antibody	Thermo Fisher Scientific	Cat# A-11122; RRID: AB_221569
Rabbit polyclonal anti-TDP-43 antibody	Thermo Fisher Scientific	Cat# PA5-27221; RRID: AB_2544697
Rabbit polyclonal anti-AGK antibody	Atlas Antibodies	Cat# HPA020959; RRID: AB_1854206
Rabbit monoclonal anti-Cyclophilin F [EPR11311-121] antibody	Abcam	Cat# ab231155
Rabbit polyclonal anti-MAVS (Rodent specific) antibody	Cell Signaling Technology	Cat# 4983; RRID: AB_823566
Rabbit monoclonal anti-Histone H3 (D1H2) antibody	Cell Signaling Technology	Cat# 4499; RRID: AB_10544537
Rabbit monoclonal anti-GAPDH (14C10) antibody	Cell Signaling Technology	Cat# 2118; RRID: AB_561053
Mouse monoclonal anti-ATP5A antibody [15H4C4]	Abcam	Cat# ab14748
Mouse monoclonal anti-PKR (B-10) antibody	Santa Cruz Biotechnology	Cat# sc-6282; RRID: AB_628150
Mouse monoclonal anti-cGAS (D-9) antibody	Santa Cruz Biotechnology	Cat# sc-515777; RRID: AB_2734736
Rat monoclonal anti-FLAG (9H1) antibody	In-House	N/A
Mouse monoclonal anti-β-Actin (C4) antibody	Santa Cruz Biotechnology	Cat# sc-47778 HRP; RRID: AB_2714189
Mouse monoclonal anti-DNA (AC-30-10) antibody	Progen	Cat# 61014; RRID: AB_2750935
Rabbit monoclonal anti-TOMM20 [EPR15581-54] Alexa Fluor 647 antibody	Abcam	Cat# ab209606
Rabbit polyclonal anti-TIMM44 antibody	Sigma-Aldrich	Cat# HPA043052; RRID: AB_10795713
Goat anti-Rat IgG H&L Alexa Fluor 405 antibody	Abcam	Cat# ab175671
Goat anti-Mouse IgG (H+L) Cross-Adsorbed Secondary Antibody Alexa Fluor 488 antibody	Thermo Fisher Scientific	Cat# A-11001; RRID: AB_25344069
Goat anti-Rabbit IgG (H+L) Cross-Adsorbed Secondary Antibody Alexa Fluor 568 antibody	Thermo Fisher Scientific	Cat# A-11011; RRID: AB_143157
Rabbit polyclonal anti-MNX1 (HB9) antibody	Millipore	Cat# ABN174; RRID: AB_27322012
Goat polyclonal anti-Choline Acetyltransferase antibody	Millipore	Cat# AB144P; RRID: AB_2079751
Mouse monoclonal anti-βIII Tubulin (5G8) antibody	Promega	Cat# G7121; RRID: AB_430874
Rat monoclonal anti-Myc tag [9E10] antibody	Abcam	Cat# ab206486
Anti-FLAG M2 Affinity Gel antibody	Sigma Aldrich	Cat# A2220; RRID: AB_10063035

**Biological Samples**		

Human post-mortem spinal cord tissues (sALS and MS)	Victorian Brain Bank	Table S1

**Chemicals, Peptides, and Recombinant Proteins**		

FuGENE HD Transfection Reagent	Promega	Cat# E2311
Lipofectamine 2000 Transfection Reagent	Thermo Fisher Scientific	Cat# 11668030
Ethidium bromide	Sigma-Aldrich	Cat# E8751; CAS 1239-45-8
Uridine	Sigma-Aldrich	Cat# U3003; CAS 58-96-8
Geneticin Selective Antibiotic (G418 Sulfate)	Thermo Fisher Scientific	Cat# 11811023; CAS 108321-42-2
Doxycycline hyclate	Sigma-Aldrich	Cat# D9891; CAS 24390-14-5
Blasticidin	InvivoGen	Cat# ant-bl-1
Poly(dA:dT)	InvivoGen	Cat# tlrl-patn; CAS 86828-69-5
Deoxyribonucleic acid sodium salt from herring testes (HT-DNA)	Sigma-Aldrich	Cat# D6898; CAS 438545-06-3
2′3′-c-di-AM(PS)2(Rp, Rp)	InvivoGen	Cat# tlrl-nacda2r; CAS 1638750-95-4
ABT-737	Active Biochemicals	Cat# A-1002; CAS 852808-04-9
MitoSOX™ Red	Thermo Fisher Scientific	Cat# M36008
Tetramethylrhodamine methyl ester perchlorate (TMRM)	Thermo Fisher Scientific	Cat# T5428; CAS 115532-50-8
Cyclosporin A	Sigma-Aldrich	Cat# C3662; CAS 59865-13-3
RU.521 (cGAS inhibitor)	M. Ascano Laboratory	Vincent et al., 2017
H-151 (STING inhibitor)	Life Chemicals	Haag et al., 2018
Mitoquinol	Cayman Chemical	Cat# 89950; CAS 845959-55-9
MitoTEMPO (hydrate)	Cayman Chemical	Cat# 16621; CAS 1569257-94-8
TDP-43 inhibitor peptide PM1 (YGRKKRRQRRRAQFPGACGL)	This paper	Wang et al., 2016
TDP-43 control peptide CM1 (YGRKKRRQRRRAAQGFGCPL)	This paper	Wang et al., 2016
Primocin	InvivoGen	Cat# ant-pm-1
Y-27632 (RHO/ROCK pathway inhibitor)	STEMCELL Technologies	Cat# 72304; CAS 129830-38-2
CHIR99021 (GSK3 inhibitor)	STEMCELL Technologies	Cat# 72054; CAS 252917-06-9
Dorsomorphin (BMP and AMPK inhibitor)	STEMCELL Technologies	Cat# 72102; CAS 866405-64-3
SB431542 Hydrate (Activin/BMP/TGF-b pathway inhibitor)	STEMCELL Technologies	Cat# 72234; CAS 301836-41-9
All trans-Retinal	Sigma-Aldrich	Cat# R2500; CAS 116-31-4
Purmorphamine (Hedgehog pathway activator)	STEMCELL Technologies	Cat# 72202; CAS 483367-10-8
L-Ascorbic acid	Sigma-Aldrich	Cat# A4403; CAS 50-81-7
Valproic Acid (Sodium Salt)	STEMCELL Technologies	Cat# 72292; CAS 1069-66-5
Compound E	STEMCELL Technologies	Cat# 73954; CAS 209986-17-4
Paraformaldehyde 16% aqueous solution	ProSciTech	Cat# C004; CAS 30525-89-4
Glutaraldehyde solution	Sigma-Aldrich	Cat# 340855; CAS 111-30-8
VBIT-4 (VDAC1 oligomerization inhibitor)	MedChemExpress	Cat# HY-129122; CAS 2086257-77-2
Digitonin	Sigma-Aldrich	Cat# D141; CAS 11024-24-1
cOmplete Protease Inhibitor Cocktail	Roche Biochemicals	Cat# 11836145001
IGF1 recombinant human protein	Thermo Fisher Scientific	Cat# PHG0078
RPMI-1640	In-House	N/A
Dulbecco’s Modified Eagle Medium (DMEM)	Thermo Fisher Scientific	Cat# 11965092
DME/KELSO Medium	In-House	N/A
Hank’s Balanced Salt Solution (HBSS)	Thermo Fisher Scientific	Cat# 14170112
Matrigel hESC-Qualified Matrix	Corning	Cat# 354277
mTeSR™1	STEMCELL Technologies	Cat# 85850
ReLeSR	STEMCELL Technologies	Cat# 05872
KnockOut DMEM/F-12	Thermo Fisher Scientific	Cat# 12660012
Neurobasal Medium	Thermo Fisher Scientific	Cat# 21103049
N-2 Supplement (100X)	Thermo Fisher Scientific	Cat# 17502048
B-27 Supplement (50X)	Thermo Fisher Scientific	Cat# 17504044
Accutase cell detachment solution	STEMCELL Technologies	Cat# 07920
TrypLE Express Enzyme	Thermo Fisher Scientific	Cat# 12604013
Normal Goat Serum (10%)	Thermo Fisher Scientific	Cat# 50062Z

**Critical Commercial Assays**		

QuikChange Lightning Mutagenesis Kit	Agilent Technologies	Cat# 210513
Lactate Dehydrogenase Activity Assay Kit	Sigma-Aldrich	Cat# MAK066
VeriKine-HS Mouse Interferon Beta Serum ELISA Kit	PBL Assay Science	Cat# 42410-1
VeriKine-HS Human Interferon Beta Serum ELISA Kit	PBL Assay Science	Cat# 41415-1
Human CXCL10/IP-10 Quantikine ELISA kit	R&D Sysytems	Cat# SIP100
2′3′-cGAMP ELISA Kit	Cayman Chemical	Cat# 501700

**Deposited Data**		

Original blots	This paper	Mendeley Data: https://doi:10.17632/kx9v83c65r.1

**Experimental Models: Cell Lines**		

Human embryonic kidney (HEK) 293T	ATCC	CRL-3216
AGK^Flp-In^ control HEK293T	Diana Stojanovski Laboratory	Kang et al., 2017
AGK^−/−^ HEK293T	Diana Stojanovski Laboratory	Kang et al., 2017
AGK^−/− +WT^ HEK293T	Diana Stojanovski Laboratory	Kang et al., 2017
AGK^−/− +G126E-3XFLAG^ HEK293T	Diana Stojanovski Laboratory	Kang et al., 2017
THP-1 human monocytic cells (sex: male, age: 1)	ATCC	TIB-202
THP-1 Cas9-mCherry cells	This paper	N/A
THP-1 pSLIK-neo cells	This paper	N/A
THP-1 pSLIK-TDP-43 WT cells	This paper	N/A
THP-1 pSLIK-TDP-43 Q331K cells	This paper	N/A
THP-1 STING^CRISPR−/−^ cells	This paper	N/A
THP-1 STING^CRISPR−/−^ pSLIK-neo cells	This paper	N/A
THP-1 STING^CRISPR−/−^ pSLIK-TDP-43 WT cells	This paper	N/A
THP-1 STING^CRISPR−/−^ pSLIK-TDP-43 Q331K cells	This paper	N/A
WT Immortalized mouse embryonic fibroblasts (MEFs) (sex: ND)	This paper	N/A
MAVS^−/−^ MEFs (sex: ND)	Sandra Nicholson Laboratory	Nguyen et al., 2017
cGAS^−/−^ MEFs (sex: ND)	Benjamin Kile Laboratory	White et al., 2014
STING^−/−^ MEFs (sex: ND)	Benjamin Kile Laboratory	White et al., 2014
Bak^−/−^/Bax^−/−^ MEFs (sex: ND)	Benjamin Kile Laboratory	White et al., 2014
Vdac1^−/−^/Bak^−/−^ MEFs (sex: ND)	Grant Dewson Laboratory	Chin et al., 2018
Vdac1^−/−^/Bax^−/−^ MEFs (sex: ND)	Grant Dewson Laboratory	Chin et al., 2018
PKR^−/−^ MEFs (sex: ND)	Anthony Sadler Laboratory	Irving et al., 2012
Mcl1^−/−^ Cas9/Blasticidin MEFs	This paper	N/A
Mcl1^−/−^ pSLIK-neo MEFs	This paper	N/A
Mcl1^−/−^ pSLIK-hTDP-43 WT MEFs	This paper	N/A
Mcl1^−/−^ pSLIK-hTDP-43 Q331K MEFs	This paper	N/A
Ppid^CRISPR−/−^ pSLIK-neo MEFs	This paper	N/A
Ppid^CRISPR−/−^ pSLIK-hTDP-43 WT MEFs	This paper	N/A
Ppid^CRISPR−/−^ pSLIK-hTDP-43 Q331K MEFs	This paper	N/A
Mouse motor neuron-like hybrid cells NSC-34 (sex: ND)	Cellutions Biosystems	CLU140
pSLIK-neo NSC-34	This paper	N/A
pSLIK-hTDP-43 WT NSC-34	This paper	N/A
pSLIK-hTDP-43 Q331K NSC-34	This paper	N/A
Human iPSC healthy control HC1 (NCRM-1) (male, cord blood)	National Institute of Neurological Disorders and Stroke	Efthymiou et al., 2015
Human iPSC healthy control HC2 (NCRM-5) (male, cord blood)	National Institute of Neurological Disorders and Stroke	Efthymiou et al., 2015
Human iPSC healthy control HC3 (WT1) (sex: ND, age: 8)	Alessandro Rosa Laboratory	Lenzi et al., 2015
Human iPSC healthy control HC4 (female, age: 62)	This paper	N/A
Human iPSC healthy control HC5 (male, age: 74)	This paper	N/A
Human iPSC healthy control HC6 (female, age: 88)	This paper	N/A
Human iPSC ALS-TARDBP G298S (TALSTDP-47.10) (male, age: 43)	Target ALS Foundation	RRID: CVCL_FA03
Human iPSC ALS-TARDBP M337V (CiRA00026) (female, age: 62)	Cell Bank Riken BioResource Research Center	RRID: CVCL_T783 (Egawa et al., 2012)
Human iPSC ALS-TARDBP A382T (male, age: 50)	Alessandro Rosa Laboratory	Lenzi et al., 2015
Human iPSC ALS-C9ORF72 HRE 1 (female, age: 56)	This paper	N/A
Human iPSC ALS-C9ORF72 HRE 2 (male, age: 59)	This paper	N/A
Human iPSC ALS-C9ORF72 HRE 3 (male, age: 52)	This paper	N/A
Human iPSC ALS-SOD1 1 (G93A) (male, age: 47)	This paper	N/A
Human iPSC ALS-SOD1 2 (I114T) (sex: male, age: 62)	This paper	N/A
Human iPSC ALS-SOD1 3 (G85S) (sex: female, age: 29)	This paper	N/A
Mouse primary bone marrow-derived macrophages (WT) (sex: male, age d150)	This paper	N/A
Mouse primary bone marrow-derived macrophages (Prp-TDP-43^Tg/+^) (sex: male, age d150)	This paper	N/A

**Experimental Models: Organisms/Strains**		

Mouse: C57BL/6J (WT)	Jackson Laboratory	Cat# 000664
Mouse: B6.Cg-Tg(Prnp-TARDBP^∗^A315T)95Balo/J (Prp-TDP-43^Tg/+^)	Jackson Laboratory	Cat# 010700
Mouse: Prp-TDP-43^Tg/+^ x *Sting*^+/−^	This paper	N/A
Mouse: Prp-TDP-43^Tg/+^ x *Sting*^−/−^	This paper	N/A

**Oligonucleotides**		

Genotyping: Human *TARDBP* (A315T) forward	https://www.jax.org/Protocol?stockNumber=010700&protocolID=26362	GGATGAGCTGCGGGAGTTCT
Genotyping: Human *TARDBP* (A315T) reverse	https://www.jax.org/Protocol?stockNumber=010700&protocolID=26362	TGCCCATCATACCCCAACTG
Genotyping: Mouse *Sting* forward	This paper	GCTGGGAATTGAACGTAGGA
Genotyping: Mouse *Sting* reverse	This paper	GAGGAGACAAAGGCAAGCAC
Genotyping: Mouse *Sting* KO forward	This paper	GTGCCCAGTCATAGCCGAAT
Human *TARDBP* A315T mutagenesis forward	This paper	GGATTAATGCTGAA CGTACCAAAGTTCATCCCACCA
Human *TARDBP* A315T mutagenesis reverse	This paper	TGGTGGGATGAACT TTGGTACGTTCAGCATTAATCC
Human *TARDBP* Q331K mutagenesis forward	This paper	CCCCAACTGCTCTT TAGTGCTGCCTGGGC
Human *TARDBP* Q331K mutagenesis reverse	This paper	GCCCAGGCAGCACT AAAGAGCAGTTGGGG
Human *STING* sgRNA forward	This paper	AGAGCACACTCTCCGGTACC
Human *STING* sgRNA reverse	This paper	GGTACCGGAGAGTGTGCTCT
Mouse *Ppid* sgRNA forward	This paper	CGTGCCAAAGACTGCAGGTA
Mouse *Ppid* sgRNA reverse	This paper	TACCTGCAGTCTTTGGCACG
qPCR: Human *IFNB1* forward	Balka et al., 2020	TGTCGCCTACTACCTGTTGTGC
qPCR: Human *IFNB1* reverse	Balka et al., 2020	AACTGCAACCTTTCGAAGCC
qPCR: Human *TNF* forward	Balka et al., 2020	TCTCTCAGCTCCACGCCATT
qPCR: Human *TNF* reverse	Balka et al., 2020	CCCAGGCAGTCAGATCATCTTC
qPCR: Human *MNX1* forward	This paper	GCACCAGTTCAAGCTCAAC
qPCR: Human *MNX1* reverse	This paper	GCTGCGTTTCCATTTCATCC
qPCR: Human *CHAT* forward	This paper	AGCCTCATCTCTGGTGTACTC
qPCR: Human *CHAT* reverse	This paper	GCCCATAGTATTGCTTCATGC
qPCR: Human *HPRT* forward	Balka et al., 2020	TCAGGCAGTATATCCAAAGATGGT
qPCR: Human *HPRT* reverse	Balka et al., 2020	AGTCTGGCTTATATCCAACACTTCG
qPCR: Mouse *Ifnb1* forward	Balka et al., 2020	CCAGCTCCAAGAAAGGACGA
qPCR: Mouse *Ifnb1* reverse	Balka et al., 2020	TGGATGGCAAAGGCAGTGTA
qPCR: Mouse *Ifna6* forward	This paper	GCTTTCCTGATGGTTTTGGTG
qPCR: Mouse *Ifna6* reverse	This paper	AGGCTTTCTTGTTCCTGAGG
qPCR: Mouse *Ifit1* forward	This paper	AGAGTCAAGGCAGGTTTCTG
qPCR: Mouse *Ifit1* reverse	This paper	TGTGAAGTGACATCTCAGCTG
qPCR: Mouse *Mx1* forward	This paper	GATCCGACTTCACTTCCAGATGG
qPCR: Mouse *Mx1* reverse	This paper	CATCTCAGTGGTAGTCAACCC
qPCR: Mouse *Irf7* forward	This paper	AAGCTGGAGCCATGGGTATG
qPCR: Mouse *Irf7* reverse	This paper	CGATGTCTTCGTAGAGACTGTTGG
qPCR: Mouse *Tnf* forward	Balka et al., 2020	CCAAATGGCCTCCCTCTCAT
qPCR: Mouse *Tnf* reverse	Balka et al., 2020	TGGTGGTTTGCTACGACGTG
qPCR: Mouse *Il1b* forward	Balka et al., 2020	TTGACGGACCCCAAAAGATG
qPCR: Mouse *Il1b* reverse	Balka et al., 2020	CAGCTTCTCCACAGCCACAA
qPCR: Mouse *Il6* forward	Balka et al., 2020	CCAGAAACCGCTATGAAGTTCC
qPCR: Mouse *Il6* reverse	Balka et al., 2020	CGGACTTGTGAAGTAGGGAAGG
qPCR: Mouse *Hprt* forward	Balka et al., 2020	TGAAGTACTCATTATAGTCAAGGGCA
qPCR: Mouse *Hprt* reverse	Balka et al., 2020	CTGGTGAAAAGGACCTCTCG
DNA assay: Human *MT-ND1* forward	This paper	CTCTTCGTCTGATCCGTCCT
DNA assay: Human *MT-ND1* reverse	This paper	TGAGGTTGCGGTCTGTTAGT
DNA assay: Human *MT-ND2* forward	This paper	GTAGACAGTCCCACCCTCAC
DNA assay: Human *MT-ND2* reverse	This paper	TTGATCCCGTTTCGTGCAAG
DNA assay: Human *POLG1* forward	This paper	CTGCCATAAGGTCTGCAGGT
DNA assay: Human *POLG1* reverse	This paper	CTCCTTTCCGTCAACAGCTC
DNA assay: Human *L1ORF1* gDNA forward	Papasotiriou et al., 2017	AGAACGCCACAAAGATACTCCTCG
DNA assay: Human *L1ORF1* gDNA reverse	Papasotiriou et al., 2017	CTCTCTTCTGGCTTGTAGGGTTTCTG
DNA assay: Mouse *Nd1* forward	White et al., 2014	CAAACACTTATTACAACCCAAGAACA
DNA assay: Mouse *Nd1* reverse	White et al., 2014	TCATATTATGGCTATGGGTCAGG
DNA assay: Mouse *Nd2* forward	White et al., 2014	CCATCAACTCAATCTCACTTCTATG
DNA assay: Mouse *Nd2* reverse	White et al., 2014	GAATCCTGTTAGTGGTGGAAGG
DNA assay: Mouse *L1* gDNA forward	Newman et al., 2012	TAGGAAATTAGTTT GAATAGGTGAGAGGGT
DNA assay: Mouse *L1* gDNA reverse	Newman et al., 2012	TCCAGAAGCTGTCAGGTTCTCTGGC
DNA assay: *RNA18S* gDNA forward	Chang et al., 2013	GTAACCCGTTGAACCCCATT
DNA assay: *RNA18S* gDNA reverse	Chang et al., 2013	CCATCCAATCGGTAGTAGCG

**Recombinant DNA**		

pSLIK-Neo	Aaron D. Gitler Laboratory	Armakola et al., 2012
pSLIK-hTDP-43	Aaron D. Gitler Laboratory	Armakola et al., 2012
pSLIK-hTDP-43 Q331K	Aaron D. Gitler Laboratory	Armakola et al., 2012
pGW1-hTDP-43-EGFP	Steven Finkbeiner Laboratory	Armakola et al., 2012
pGW1-hTDP-43 A315T-EGFP	This paper	N/A
pGW1-hTDP-43 Q331K-EGFP	This paper	N/A
pMIH-FLAG-mmcGAS	Benjamin Kile Laboratory	White et al., 2014

**Software and Algorithms**		

Fiji	https://fiji.sc/	RRID: SCR_002285
Image Lab	https://www.bio-rad.com/en-us/sku/1709690-image-lab-software	RRID: SCR_014210
Prism 8	https://www.graphpad.com/	RRID: SCR_002798
Imaris	https://imaris.oxinst.com/packages	RRID: SCR_007370
Adobe Illustrator	https://www.adobe.com/products/illustrator.thml	RRID: SCR_010279
CaseViewer	https://www.3dhistech.com/solution/caseviewer/	RRID: SCR_017654
FlowJo v.10	https://www.flowjo.com/solutions/flowjo	RRID: SCR_008520
MouseMove	Samson et al., 2015	N/A

### Resource Availability

#### Lead Contact

Lead contact is Seth L. Masters (masters@wehi.edu.au).

#### Materials Availability

Further information and requests for resources and reagents listed in Key Resources Table should be directed to the Lead Contact.

#### Data and Code Availability

Original western blots for the main figures and supplemental figures are available at Mendeley Data (https://doi:10.17632/kx9v83c65r.1).

### Experimental Model and Subject Details

#### Animal

Mice transgenic for the human TDP-43 mutant allele A315T have been described previously (Wegorzewska et al., 2009) (B6.Cg-Tg(Prnp-TARDBP^∗^A315T)95Balo/J, JAX stock no.:010700, referred to as Prp-TDP-43^Tg/+^). Prp-TDP-43^Tg/+^ mice were backcrossed for at least ten generations and then maintained on a C57BL/6 background. *Sting* (Jin et al., 2011) knockout strains have been described previously. *Sting*^+/−^ mice on the congenic C57BL/6 background were crossed with Prp-TDP-43^Tg/+^ mice to create Prp-TDP-43^Tg/+^*Sting*^+/−^, and further crossed with other non-littermate mice of the same genotype in order to generate the offspring of Prp-TDP-43^T/+^*Sting*^+/+^, Prp-TDP-43^Tg/+^*Sting*^+/−^ and Prp-TDP-43^Tg/+^*Sting*^−/−^. All animals analyzed in this study were males. Care of the Prp-TDP-43^Tg/+^ male mice was adapted from previously described methods (Becker et al., 2017). In addition to regular chow, all male mice were given DietGel Boost (ClearH_2_O) in a cup on the floor of the cage from day 30 until the experiment endpoint to ensure that the impaired mice could easily access their food and water. The mice were weighed and visually checked for an ALS phenotype by animal technicians daily until they reached the euthanasia endpoint of severe motor dysfunction (see gait impairment scoring). Animal motor assessment was conducted in mice aged 120-130 days. Mice were genotyped using primers listed in the Key Resources Table. Animal procedures were approved by the Walter and Eliza Hall Institute Animal Ethics Committee (Ethics application: 2017.029).

#### Immortalized cell lines

Immortalized mouse embryonic fibroblasts (MEFs) lacking MAVS (Nguyen et al., 2017), PKR (Irving et al., 2012), Vdac1 (Chin et al., 2018), cGAS, Sting, Bak/Bax, Mcl1 or WT control were described previously (White et al., 2014). (Gifts: MAVS^−/−^ from Sandra Nicholson Laboratory; PKR^−/−^ from Anthony Sadler Laboratory; Vdac1^−/−^ from Grant Dewson Laboratory; others from Benjamin Kile Laboratory). These MEF lines were maintained in DME/KELSO medium (in-house DMEM containing 40 mM sodium bicarbonate, 1 mM HEPES, 0.0135 mM folic acid, 0.24 mM L-asparagine, 0.55 mM L-arginine, 1x Pen/Strep and 22.2 mM D-glucose) supplemented with 10% fetal bovine serum (FBS) (Sigma-Aldrich). Flp-In control, *AGK*^*-/*-^, *AGK*^−/− +WT^, *AGK*^−/− +G126E^ HEK293T were gifts from Diana Stojanovski Laboratory (Kang et al., 2017), WT HEK293T cells (ATCC) and NSC-34 cells (Cellutions Biosystems) were maintained in complete Dulbecco’s Modified Eagle Medium (DMEM containing 10% FBS, 1% D-glucose, 0.11% sodium pyruvate, 0.1% streptomycin and 100U/mL penicillin). Human monocytic THP-1 cells (ATCC) were grown in complete RPMI-1640. The above cell culture was conducted at 37°C in a humidified atmosphere with 10% CO_2_. See the Key Resources Table for further information of the cell lines used in this study.

#### Primary macrophages culture

Bone marrow was extracted from femurs of 150 day old WT and Prp-TDP-43^T/+^ mice (all males) for *ex vivo* differentiation of macrophages (BMDMs) in complete DMEM supplemented with 20% L929 conditioned medium (LCM) for 6 days at 37°C in a humidified atmosphere with 10% CO_2_. On day 6, cells were detached and re-seeded in complete DMEM supplemented with 10% LCM overnight prior to further experiments.

#### Human induced pluripotent stem cell (iPSC)

The established human iPSC lines used in this study were derived from six controls, three patients carrying mutations in *TARDBP* (G298S, M337V and A382T), three patients with repeat expansions in *C9ORF72* and three patients carrying mutations in *SOD1* (G85S, G93A and I114T). All iPSCs were maintained on Matrigel-coated 6-well plates in mTeSR1 containing 1x Primocin and passaged 1:6 using ReLeSR with ROCK inhibitor Y-27632 for the first 24 hours at 37°C in a humidified atmosphere with 5% CO_2_. The medium was replaced daily. Cells were cyropreserved in mTeSR1/10% DMSO. See the Key Resources Table for further information of the iPSC lines used in this study.

#### Human post-mortem tissue

Spinal cord samples of patients with ALS (n = 16) and multiple sclerosis (MS) (n = 12) were recruited from the Victorian Brain Bank and analyzed for cGAMP levels (see Table S1). Approval to use post-mortem human tissue was granted by the University of Melbourne Human Ethics Committee (approval numbers 1238124 and 1750665).

#### Mitochondrial DNA-depleted cells (ρ^0^)

Depletion of mtDNA was performed in a relevant culture medium containing 100 ng/mL ethidium bromide, 100 μg/mL sodium pyruvate and 50 μg/mL uridine as previously demonstrated (Hashiguchi and Zhang-Akiyama, 2009). THP-1 cells were cultured in this medium for 3 weeks and then rested for an additional 2 weeks in the presence of uridine to achieve mtDNA-depleted cells. Human iPSC-derived motor neurons were treated in a similar way, except a lower dose of 50 ng/mL ethidium bromide was used. The depletion was analyed using real-time qPCR to measure expression of mitochondrial genes or nuclear genes. The primer sequences used are provided in the Key Resources Table.

#### CRISPR/Cas9-mediated gene deletion

We generated *STING*^CRISPR−/−^ THP-1 cells and *Ppid*^CRISPR−/−^ MEFs as described previously (Baker and Masters, 2018). Third generation lentiviral transduction was performed to generate cells expressing Cas9 fused to mCherry or blasticidin ressitance gene, which were subjected to positive selection via FACS sorting or antibiotic treatment for 2 weeks respectively. Doxycycline-inducible sgRNAs were cloned using a pFgH1tUT (BFP tagged) plasmid and subsequently transduced into the target cells expressing Cas9. Cells were treated with doxycycline for 72 hours and then rested for an additional 48 hours prior to experiments. Gene disruption was confirmed by immunoblot analysis of target proteins and functional analysis. The targeting guide sequences are provided in the Key Resources Table. Gene deletion was then confirmed by western blot and functional analysis.

### Method Details

#### Lentiviral transduction

Third generation lentiviral constructs including pSLIK-Neo (vector), hTDP-43 WT and Q331K were used to generate lentivirus as described (Balka et al., 2020; Moghaddas et al., 2018). HEK293T cells were transiently transfected with pSLIK plasmids, pMDL (packaging), RSV-REV (packaging) and VSVg (envelope) using Lipofectamine 2000 diluted in OptiMEM (Thermo Fisher Scientific) to generate lentiviral particles. The cell culture supernatant was collected 48 hours later and filtured through 0.45 mm filteres prior to transduction, for which 5x10^5^ target cells were centrifuged with the lentivirus in the presence of polybrene (Sigma-Aldrich) at 839 x g for 3 hours at 32°C and cultured at 37°C overnight. Transduced cells were subsequently subjected to antibiotic selection with G418 (Thermo Fisher Scientific) to generate stable cell lines carrying doxycycline-inducable 3xFLAG-tagged (N’) and Myc-tagged (C’) TDP-43.

#### Plasmid mutagenesis

The constructs pGW1-hTDP-43 A315T or Q331K obtained via site-directed mutagenesis using the QuikChange Lightning Kit (Agilent Technologies) using the oligonucleotide primers listed in the Key Resources Table. The mutagenesis was confirmed via Sanger Sequencing.

#### Cell transfection

Transfection of MEFs was performed using FuGENE HD (Promega) at a transfection reagent:DNA ratio of 3:1 for 48 hours. For HEK293T cells, Lipofectamine 2000 (Life Technologies) was used for 24 hours according to manufacturer’s instructions.

#### Generation of iPSC-derived motor neuron progenitors (MNPs)

Differentiation of iPSCs into MNPs was performed as described previously (Du et al., 2015). Human iPSCs were cultured in a chemically defined Neural Medium: DMEM/F12:Neurobasal (1:1) supplemented with 0.5x N-2, 0.5x B-27, 0.1mM L-ascorbic acid, 1x Glutamax and 1x Primocin containing 3 μM CHIR99021, 2 μM Dorsomorphin and 2 μM SB431542 37°C in a humidified atmosphere with 5% CO_2_ for 6 days for induction of neuroepithelial progenitors (NEPs). The NEPs were dissociated with ReLeSR as per manufacturer’s instruction and cultured 1:6 on Matrigel-coated plates in the Neural Medium containing 1 μM CHIR99021, 2 μM Dorsomorphin and 2 μM SB431542 for 6 days for induction of MNPs. Y-27632 was used for the first 24 hours, and the medium was changed every other day. At this stage of differentiation, MNPs were either expanded in the Neural Medium containing 3 μM CHIR99021, 2 μM Dorsomorphin, 2 μM SB431542, 0.1 μM all-trans retinoic acid (RA), 0.5 μM Purmorphamine (Pur) and 0.5 mM Valproic Acid prior to differentiation of motor neuron or cryopreserved in the same medium containing additional 10% DMSO. All medium and reagents used are listed in the Key Resources Table.

#### Differentiation of motor neurons (MNs)

The MNPs were dissociated with Accutase and cultured on Matrigel-coated plates in the Neural Medium containing 0.5 μM all-trans RA and 0.1 μM Pur for 6 days into premature MNX1^+^ MN. Y-27632 was used for the first 24 hours. Subsequently, cells were detached with Accutase to generate a single cell suspension and matured in the medium supplemented with 0.1 μM Compound E for 10 days into ChAT^+^ MNs. The medium was replaced every other day for both stages of differentiation. Cell markers of motor neurons, including MNX1, ChAT and βIII-Tubulin, were confirmed using an inverted SP8 confocal microscopy (Leica) or quantitative real-time PCR. The antibodies and primers used are provided in the Key Resources Table.

#### Super resolution microscopy

MEF cell lines expressing Dox-induced FLAG-tagged TDP-43 were seeded onto glass coverslips (18mm x 18mm, thickness 1½, Zeiss) for the indicated times and prepared for imaging as previously described (McArthur et al., 2018). In brief, cells were fixed in ice-cold methanol/acetone containing 0.1% Glutaraldehyde, blocked and incubated in 3% normal goat serum/0.1% Triton X-100 overnight at 4°C with primary antibodies as follows: anti-FLAG tag, anti-DNA and anti-TIM44 in blocking buffer. Following two washes in 0.1% Triton X-100/PBS and one hour incubation with secondary antibodies (goat anti-rabbit AF568, goat anti-mouse AF488 and goat anti-rat AF405). After an additional probing with anti-TOM20 AF647 overnight at 4°C, coverslips were mounted onto the microscopy slide using Prolong Diamond Antifade Mountant (Thermo Fisher Scientific). HEK293T cells were seeded onto poly-L-lysine-coated coverslips and stained with anti-Myc tag and other antibodies listed above. Four color imaging was performed on the OMX-SR system (GE Healthcare) using a 60Å∼1.42 NA oil immersion lens (Olympus). Three-dimensional surface construction and zoom-in snapshots of selected regions were created using Imaris software (v9.12). For quantification, the nuclear region was removed and any cells at the edge of the ROI was cropped out, surfaces were created for the DNA and TDP-43 channels. This approach allows spatial calculation of the proportion of DNA or TDP-43 regions overlapped or dissociated with the mitochondrial channels, such as TIM44 and TOM20, using Imaris software.

#### Administration of STING inhibitor *in vivo*

At day 110, Prp-TDP-43^Tg/+^ mice were injected with H-151 (210 μg) or an equivalent amount of DMSO in 200 μL PBS+10% Tween80 by intraperitoneal injection three times a week for a total 4-week treatment course. To minimize the impact, IP injections were rotated around the 4 quadrants of the abdomen, so that the same region was only injected twice, 4 days apart. No adverse reactions were observed during the treatment.

#### Animal phenotype scoring

We collected data for gait impairment in each mouse line using adapted or previously described methods (Becker et al., 2017). Scoring was performed blinded to the genotype twice a week by animal technicians until the humane euthanasia end point or at day 300. Taken briefly, a score of 0 was given to the mouse with no motor impairment; a score of 1 was given to the mouse with a tremor while walking; a score of 2 was given to the mouse displaying a lowered pelvis and swimming gait while moving forward; a score of 3 was given to the mouse struggling to move forward and dragging its abdomen on the ground; a score of 4 marked the euthanasia end point in which the mouse failed to upright itself within 30 s. The scoring was interpreted for the slope of the linear regression across the lifespan per mouse, indicating progression of ALS-associated motor dysfunction.

#### Animal motor assessment

##### The Rotarod test

Motor co-ordination and balance was measured using a rotating rod (Rotamex-5, Columbus Instruments). It measured the time (latency) it takes the mouse to fall off the apparatus accelerating from 4 to 40 rpm in 288 s (1 rpm/8 s). All mice at day 120-130 received a 3-day training course with three trials a day prior to the assay. On the day of testing, mice were kept in their cages and acclimatised to the procedure room for at least 15 minutes. The test phase consists of three trials separated by 15-minute intervals to avoid habituation, and the average of three trials was taken into data analysis.

##### Open Field (OF) test

To quantify differences in generalized locomotor activity, mice were subjected to the OF test as previously described (Samson et al., 2015). In brief, mice at day 120-130 were placed in the center of a custom-built circular arena with a white melamine floor (diameter: 90cm) and black plastic wall (height: 39cm). This was performed in a quiet (∼50 dB) and dimly lit (∼35 lux) room. The OF was wiped clean with 70% ethanol and allowed to dry between test sessions to minimize olfactory cues. The movement of each mouse in the OF was video captured using an overhead HD C615 webcam (Logitech) and then a detailed analysis of movement was performed using Fiji and MouseMove (Samson et al., 2015).

#### Mouse CNS tissue collection

Brains and spinal cords were collected from mice following cardiac perfusion with PBS. For cytokine profiling, tissues were homogenized using metal beads at 30Hz for 90 s in 1 mL Trizol (Thermo Fisher Scientific) with a TissueLyser II (QIAGEN) then total RNA was isolated for qPCR. For immunochemistry, mice were perfused with 4% paraformaldehyde (PFA) after perfusion with PBS. Tissues were then immersed in 4% PFA for 3 days, cryoprotected and embedded for cryosection (7 μm). Cresyl violet was used to stain Nissl bodies as a marker to compare neuronal density in cortical layer V measured using the ‘Analyze Particles’ feature of Fiji and present as neurons/mm^2^.

#### Immunoblotting

Cells were lysed in 1x RIPA buffer for total lysis (20mM Tris-HCl pH 7.4, 150 mM NaCl, 1 mM EDTA, 1% Triton X-100, 10% glycerol, 0.1% SDS, 0.5% deoxycholate, 10 mM NaPPi, 5 mM NaF and 1 mM Na_3_VO_4_) or in 0.025% digitonin/MELB buffer (20 mM HEPES/KOH, pH 7.5, 250 mM sucrose, 1 mM EDTA, 50 mM KCl, 2.5 mM MgCl_2_) for cytosolic lysis unless otherwise stated in the specific figure legends. All cell lysis buffers were supplemented with 1 mM PMSF and cOmplete protease inhibitors (Roche Biochemicals). RIPA-lysed samples were processed through Pierce centrifuge columns (Thermo Fisher Scientific) to remove DNA. Following addition of reducing SDS-PAGE sample loading buffer (1.25% SDS, 12.5% glycerol, 62.5mM Tris-HCl pH 6.8, 0.005% bromophenol blue, 50mM dithiothreitol) and denaturation at 95°C for 10 min, samples were separated on Novex 4%–12% precast SDS-PAGE gels (Thermo Fisher Scientific) with MES running buffer (Thermo Fisher Scientific), and subsequently transferred onto polyvinylidene difluoride (PVDF) membrane (Millipore). Membranes were blocked in 5% skim milk in Tris-buffered saline (TBS) containing 0.1% Tween 20 before overnight incubation with specific primary antibodies at 4°C. All listed primary antibodies were used at 1:1000 unless otherwise stated in the specific figure legends. Membranes were then washed and incubated with appropriate HRP-conjugated secondary antibodies, developed immunoreactivity (Chemiluminescent HRP substrate, Millipore) and imaged using the ChemiDoc Touch Imaging System (BioRad).

#### Cytosolic DNA immunoprecipitation and subcellular fractionation

Following induction of TDP-43 for the time indicated, cell lysates were prepared similarly to a previously published protocol (West et al., 2015). Approximately 10 × 10^6^ HEK293T cells or MEFs were lysed at 4°C for 10 min with 1 mL of 0.025% digitonin/MELB buffer. Cell lysates were centrifuged twice at 13,000 x g for 5 minutes at 4°C to separate the soluble fraction from a pellet that contains the heavy membrane fraction. Human iPSC-derived motor neurons were lysed in 0.0045% digitonin/MELB buffer instead. Immunoprecipitation of FLAG-tagged mouse cGAS (pMIH-FLAG-mmcGAS) was performed using anti-FLAG M2 affinity Gel. Samples were incubated at 4°C for 2 hours on a rotator, washed extensively and then subjected to DNA extraction using a NucleoSpin Tissue XS kit (Macherey-NaGel). Quantification of coprecipitated DNA was performed similarly to previously described protocol (White et al., 2014). Alternatively, half the cells were subjected to cytoplasmic lysis (digitonin), while the other half were subjected to total cell lysis in RIPA buffer. DNA was then purified and analyzed by real-time PCR as relative expression to *Gapdh* (mouse cells) or *ACTIN* (Human cells) in total lysis, presented as the ratio of cytosolic compared to overall input of total cell lysis.

Cortex and spinal cords were collected from symptomatic Prp-TDP-43^Tg/+^ mice or healthy littermate controls following cardiac perfusion with PBS. Separate CNS tissues were passed through 100 μm nylon mesh and followed by incubation with TrypLE Express (GIBCO) at 37°C for 20 mins. Cortical and spinal cords homogenates were mechanically dissociated by trituration and passed through 70 μm nylon mesh. Cells were washed in PBS and were further subjected to 30% Percoll density gradient centrifugation at 300 x *g* at 22°C for 20 mins in order to separate myelin from viable cellular fraction. Cells were subsequently processed for subcellular fractionation and DNA analysis as described above.

#### Quantitative real-time PCR

Total RNA was isolated using the ISOLATE II RNA Mini Kit (Bioline) as per manufacturer’s instructions and reverse transcribed to cDNA using oligo(dT) nucleotides and SuperScript III Reverse Transcriptase (Thermo Fisher Scientific). Quantitative real-time PCR was performed using SYBR Green/ROX qPCR Master Mix (Thermo Fisher Scientific) on a ViiA 7 Real-Time PCR system (Thermo Fisher Scientific). Cytokine expression from qPCR is represented as gene expression relative to *Hprt/HPRT* as ΔCt. Where indicated, relative gene expression in TDP-43 WT or mutant overexpressing cells was further normalized to expression in vector control transfected/overexpressed cells of the same genotype in the same experiment and represented as fold change. Primers were designed using the Integrated DNA Technologies online tool and listed in the Key Resources Table.

#### Enzyme-Linked Immunosorbent Assay (ELISA)

Serum from the Prp-TDP-43^Tg/+^ mice or cell culture supernatants were analyzed for IFNβ and IP-10 protein for the time indicated. Levels of cGAMP in cell lysates, murine serum and CNS tissues from Prp-TDP-43^Tg/+^ mice and ALS patients were measured by 2′3′-cGAMP competitive ELISA Kit (Cayman Chemical). For sample preparation, cells were lysed in M-PER™ Mammalian Protein Extraction Reagent (Thermo Fisher Scientific) whereas CNS tissues were homogenized in 80% methanol, centrifuged at 21,000 x g for 5 minutes at 4°C to remove debris, and then the soluble fraction was freeze-dried before resuspension in PBS.

#### Detection of mitochondrial stress

Human iPSC-derived MNs were detached with Accutase to generate single cell suspension washed in 1 mL of warm Hank’s Balanced Salt Solution (HBSS) And centrifuged at 200 x g for 3 minutes at room temperature with a low brake (3). Cells were incubated with MitoSOX™ Red (mitochondrial ROS production) or TMRM dye (mitochondrial membrane potential mΔψ) as previously described (Wang et al., 2016; Zhou et al., 2011) for 10 minutes at 37°C in dark. Following three gentle washes in warm HBSS, stained neurons were resuspended in warm FACS buffer (PBS/1% FBS) and analyzed by flow cytometry (BD LSRFortessa X-20). The staining was acquired using the Y585/15 filter and analyzed for Median Fluorescence Intensity (MFI) using FlowJo software.

### Quantification and Statistical Analysis

Data are typically mean ± SEM and analyzed by t test between two groups or one- or two-way ANOVA followed by a Sidak, Tukey or Dunnett multiple comparison test as appropriate. GraphPad Prism 8 was used to generate all charts and statistical analyses. A *P value* < 0.05 was considered significant.
