# Supplementary material for: TDP-43 Triggers Mitochondrial DNA Release via mPTP to Activate cGAS/STING in ALS
Source: Cell. 2020 Oct 29;183(3):636–649.e18. doi: 10.1016/j.cell.2020.09.020 (PMC7599077; doi:10.1016/j.cell.2020.09.020)
Supplement: Document S1. Table S1 [file mmc1.pdf]

Cell, Volume *183*

Supplemental Information

TDP-43 Triggers Mitochondrial DNA Release via mPTP to Activate cGAS/STING in ALS

Chien-Hsiung Yu, Sophia Davidson, Cassandra R. Harapas, James B. Hilton, Michael J. Mlodzianoski, Pawat Laohamonthonkul, Cynthia Louis, Ronnie Ren Jie Low, Jonas Moecking, Dominic De Nardo, Katherine R. Balka, Dale J. Calleja, Fiona Moghaddas, Erya Ni, Catriona A. McLean, Andre L. Samson, Shiraz Tyebji, Christopher J. Tonkin, Christopher R. Bye, Bradley J. Turner, Genevieve Pepin, Michael P. Gantier, Kelly L. Rogers, Kate McArthur, Peter J. Crouch, and Seth L. Masters

Table S1, Related to Figure 1
Demographic characteristics of the patients.

	Amyotrophic Lateral Sclerosis (N = 16)	Multiple Sclerosis (N = 12)
Age – yr		
Median	66.2	66.5
Range	53.6 – 79.9	38.5 – 74.5
Sex – no. (%)		
Male	13 (81.3)	7 (58.3)
Female	3 (18.8)	5 (41.7)
Post-Mortem Interval - hrs		
Median	28.5	38.25
Range	7 - 56	22 - 62
ALS symptoms – no. (%)		
Bulbar	4 (25.0)	
Lower Limb	9 (56.3)	
Upper Limb	6 (37.5)	
MS symptoms – no. (%)		
Primary Progressive		4 (33.3)
Secondary Progressive		7 (58.3)
